# Tissue and cell-specific transcriptomes in cotton reveal the subtleties of gene regulation underlying the diversity of plant secondary cell walls

**DOI:** 10.1186/s12864-017-3902-4

**Published:** 2017-07-18

**Authors:** Colleen P. MacMillan, Hannah Birke, Aaron Chuah, Elizabeth Brill, Yukiko Tsuji, John Ralph, Elizabeth S. Dennis, Danny Llewellyn, Filomena A. Pettolino

**Affiliations:** 1 CSIRO Agriculture and Food, PO Box 1700, Canberra, ACT 2601 Australia; 2 Present address: Research School of Biology, The Australian National University, Canberra, ACT 2601 Australia; 3 John Curtin School of Medical Research, The Australian National University, ACT, Canberra, 2601 Australia; 4 Department of Biochemistry and the Department of Energy’s Great Lakes BioEnergy Research Center, The Wisconsin Energy Institute, 1552 University Avenue, Madison, WI 53726-4084 USA

**Keywords:** Secondary cell wall, Primary cell wall, Transcription factor, Cotton, *Gossypium hirsutum*, Cellulose synthase, Lignin, Syringyl, Guaiacyl, *p*-Hydroxyphenyl

## Abstract

**Background:**

Knowledge of plant secondary cell wall (SCW) regulation and deposition is mainly based on the Arabidopsis model of a ‘typical’ lignocellulosic SCW. However, SCWs in other plants can vary from this. The SCW of mature cotton seed fibres is highly cellulosic and lacks lignification whereas xylem SCWs are lignocellulosic. We used cotton as a model to study different SCWs and the expression of the genes involved in their formation via RNA deep sequencing and chemical analysis of stem and seed fibre.

**Results:**

Transcriptome comparisons from cotton xylem and pith as well as from a developmental series of seed fibres revealed tissue-specific and developmentally regulated expression of several NAC transcription factors some of which are likely to be important as top tier regulators of SCW formation in xylem and/or seed fibre. A so far undescribed hierarchy was identified between the top tier NAC transcription factors SND1-like and NST1/2 in cotton. Key SCW MYB transcription factors, homologs of Arabidopsis MYB46/83, were practically absent in cotton stem xylem. Lack of expression of other lignin-specific MYBs in seed fibre relative to xylem could account for the lack of lignin deposition in seed fibre. Expression of a MYB103 homolog correlated with temporal expression of SCW CesAs and cellulose synthesis in seed fibres. FLAs were highly expressed and may be important structural components of seed fibre SCWs. Finally, we made the unexpected observation that cell walls in the pith of cotton stems contained lignin and had a higher S:G ratio than in xylem, despite that tissue’s lacking many of the gene transcripts normally associated with lignin biosynthesis.

**Conclusions:**

Our study in cotton confirmed some features of the currently accepted gene regulatory cascade for ‘typical’ plant SCWs, but also revealed substantial differences, especially with key downstream NACs and MYBs. The lignocellulosic SCW of cotton xylem appears to be achieved differently from that in Arabidopsis. Pith cell walls in cotton stems are compositionally very different from that reported for other plant species, including Arabidopsis. The current definition of a ‘typical’ primary or secondary cell wall might not be applicable to all cell types in all plant species.

**Electronic supplementary material:**

The online version of this article (doi:10.1186/s12864-017-3902-4) contains supplementary material, which is available to authorized users.

## Background

Development of secondary cell walls (SCWs) was a key event during the evolution of land plants. The thick, specialised SCWs of xylem vessels and xylem fibres of angiosperms and tracheids of gymnosperms allow trees to grow to more than 100 m in height. In many species, pollen release and fertilisation, as well as seed dispersal are facilitated by specialised SCWs. Plants can also deposit localised ‘SCW-like’ structures in response to pathogen attack, or in specialised cells types such as transfer cells. Domesticated cottons produce long seed fibres that have extremely thick SCWs and hence are traded globally in textile and biomaterial markets. Our most comprehensive understanding on how SCWs are made has mainly come from detailed work over the last decade on xylem vessels and fibres in *Arabidopsis thaliana* [1–3] and other plants such as poplar [4], rice [5], grasses [6], spruce [7]. The SCWs in these plants are generally considered as ‘typical’ SCWs: they are composed of approximately equal measures of cellulose, lignins and hemicelluloses, and some proteins, but the specific composition can be variable between tissues and species. SCWs are deposited between the cell’s external primary cell wall (PCW) and the plasma membrane, often once cell expansion has ceased, and are usually orders of magnitude thicker than the PCW.

The current view of the regulation of lignocellulosic SCW deposition is that a cascade of SCW-specific NAC (for **N**AM, **A**TAF1/2, and **C**UC2) and MYB (**my**elo**b**lastosis) transcription factors (TFs) regulate downstream TFs such as other NACs, MYBs, and KNATs (**kn**otted-like from *A* *rabidopsis* *t* *haliana*) and the SCW biosynthetic genes encoding, for example, cellulose synthases (CesAs), lignin-related enzymes, enzymes for hemicellulose synthesis, and other cell wall structural components [1–3].

Different pairs of NACs act in a top tier of these lignocellulosic SCW regulatory cascades in particular Arabidopsis cell types [8, 9]. *VND6/VND7* (for **v**ascular related **N**AC **d**omain) have been shown to activate the SCW program of xylem vessels [9, 10], *SND1/NST1* (for **s**econdary wall-associated **N**AC **d**omain protein1/**N**AC **s**econdary **t**hickening promoting factor1) that of xylem fibres [11–13] and anthers [14], whereas *NST1/NST2* control the SCW program of the anther endothecium [15]. Orthologues of these genes have been identified in species such as poplar [16], rice [5], and maize [5]. The non-vascular moss *Physcomitrella patens* has VND-related NACs with roles for thick cell wall formation [17]. Some SCW NACs such as VNI1 (for **VN**D-**I**nteracting 1) and VNI2 can have repressor functions [18].

MYBs appear to play key roles as transcriptional activators in the middle tier of the SCW regulatory cascade both in angiosperms and gymnosperms. In Arabidopsis, the critical TFs are the partially redundant MYB46 and MYB83 [19–21] and this appears to also be the case in tree species [22, 23], rice, and maize [5]. The top tier NACs and MYB46/83 coordinate the expression of downstream TFs that may be activators or repressors and include XND1, SND2, SND3, KNAT7, MYB103, MYB20, MYB42, MYB43, MYB52, MYB54, MYB69, and MYB85 [24]; reviewed by [1, 9].

The genes encoding enzymes of SCW biosynthesis have been investigated in most detail in Arabidopsis, but also in woody plants [2, 25–30]. Of the ten known Arabidopsis CesAs, AtCesA4, AtCesA7, and AtCesA8 are considered important for SCW synthesis, whereas AtCesA1, AtCesA3, and AtCesA6 are essential for PCWs. Homologs of the SCW CesAs are known in many other species, such as cotton [31, 32], rice [33], poplar [34], and *Brachypodium* [35]. Other proteins/enzymes involved in cellulose biosynthesis, structure, and deposition, including in SCWs [25, 26], include COBL4 (**Cob**ra-**l**ike 4), CTL1 (**ch**i**t**inase-**l**ike protein 1), CTL2 (**c**hi**t**inase-**l**ike protein 2), TED6 (**t**racheary **e**lement **d**ifferentiation-related 6), POM2/CSI (**pom** pom 2/**c**ellulose **s**ynthase-**i**nteractive protein1), KOR (KORRIGAN), certain RLKs (receptor-like kinases) such as HERK1 (Hercules1) [36], FLA11 and FLA12 (**f**asciclin-**l**ike **a**rabinogalactan proteins 11, 12) [37], FRA1 (**fra**gile fibre 1) [38], and SuS (**su**crose **s**ynthase) [39].

Lignins are composed of syringyl (S), guaiacyl (G), and/or *p*-hydroxyphenyl (H) units derived from the phenylpropanoid pathway [40, 41]. Lignin composition varies across cell types and genera, but the enzymes of the general and lignin-specific phenylpropanoid pathway are well conserved across many species and include phenylalanine ammonia lyase (PAL), cinnamate-4-hydroxylase (C4H), 4-hydroxycinnamate CoA ligase (4CL), hydroxycinnamoyl transferase (HCT), coumarate 3-hydroxylase (C3H), caffeoyl-CoA *O*-methyltransferase (CCoAOMT), ferulate 5-hydroxylase (F5H), caffeic acid *O*-methyltransferase (COMT), cinnamoyl-CoA reductase (CCR), and cinnamyl alcohol dehydrogenase (CAD) [42, 43]. Monolignols are oxidized in the cell wall by laccases or peroxidases and form lignin in a spontaneous process [40, 43, 44]. Transcriptional activators of lignin synthesis include MYBs such as AtMYB58, AtMYB63, and PtrMYB28 [45] and other TFs. On the other hand, AtMYB4-, AtMYB7-, and AtMYB32-related TFs from different species have been shown to specifically repress lignin biosynthetic genes [46–48].

‘Typical’ SCWs also contain hemicelluloses [49], of which xylans such as glucuronoxylan (major dicot form) and glucuronoarabinoxylan (major gymnosperm and monocot form) are major components. Some genes encoding enzymes for xylan synthesis have been proposed [2].

Cotton seed fibres are long single cells surrounded by a PCW that elongates from the seed coat to reach a few centimetres in length before filling with a thick SCW. They are an exceptional example of a ‘highly cellulosic’ SCW being composed of ~94% cellulose [50–52] and with almost no lignin [53, 54]. Its SCW is so different to lignocellulosic SCWs that it is not surprising that the genes involved in its formation, or at least their expression, are different [55, 56]. On the other hand, the SCWs in cotton stem xylem are more likely to be similar to those in other plants. In a novel approach, that enables understanding of how the same genome can translate to entirely different cell walls, we have used cotton as a model to study global gene expression differences in a range of tissues and cells depositing compositionally different cell walls to understand how those compositions may be regulated at a transcriptional level. We also use state of the art NMR and biochemical analyses as well as microimaging to determine the compositional differences amongst xylem, pith, and seed fibre cells within the cotton plant. We discovered that cotton tissues contain a number of ‘atypical’ cell wall types that are regulated through modified hierarchical gene cascades that have diverged from those found in other plants.

## Methods

### Plant growth and tissue harvest

*Gossypium hirsutum* Coker 315–11 plants were grown in temperature controlled glasshouse conditions (31 °C 18 h day-time; 26 °C 6 h night-time) under natural summer daylight in Canberra, Australia. Plants were grown in pots containing soil and Osmocote fertiliser for approximately 10 weeks. Xylem and pith samples were hand-dissected from internodes 5, 6, and 7 (distance from cotyledons) from stem and flash-frozen in liquid nitrogen and stored at −80 °C; three biological replicates were harvested. Hand sections were checked using microscopy to ensure there was no cross-contamination from other tissues (Additional file 1). Seeds and attached seed fibres were harvested at 7 DPA (days post anthesis), pooled 14, 15, 16 DPA (average 15 DPA), and 25 DPA, from three biological replicates (2 bolls each), flash-frozen in liquid nitrogen, and stored at −80 °C. Seed fibres were separated from the seeds using tweezers and hand-ground to a fine powder using a mortar and pestle in liquid nitrogen. Appropriate time points for seed fibre harvest were identified based on in-house chemical analyses of a developmental series of seed fibre (Pettolino et al., submitted) and [50].

### Histology

Freshly harvested stem segments were fixed in 70% (*v*/v) ethanol and cross-sectioned to approximately 120 μm. Sections were stained with toluidine blue for 1 min and washed twice with water. Sections were viewed and photographed under a Leica DMR upright fluorescence microscope using brightfield settings.

### Cell wall polysaccharide composition from monosaccharide-linkage analysis

Ground seed fibre, pith, and xylem tissue was extracted with successive washes of 70% (*v*/v) ethanol (three times), chloroform and methanol (1:1), methanol and then acetone before drying to prepare an alcohol-insoluble residue (AIR) enriched in cell walls. AIR was de-starched using porcine pancreatic α-amylase (Sigma A3176), then carboxyl reduced (to assist in the determination of uronic acids and their methyl esters) before derivatisation by methylation, hydrolysis, reduction and acetylation according to Pettolino, Walsh [57]. Deduced monosaccharide linkages, as determined by GC-MS, were grouped according to most likely polysaccharide assignments for summation of mol% of those derivatives to give estimates of the relative proportion of each polysaccharide in the sample [57].

### Lignin analysis

Cell wall lignin content was determined as acetyl bromide lignin essentially as described [58] using alkali lignin from Aldrich (370959) as a standard. AIR was de-starched as above and protein depleted by successive washes with phosphate buffer, Triton X-100, sodium chloride, water, and acetone according to Moreira-Vilar, Siqueira-Soares [59].

**Fig. 1:**
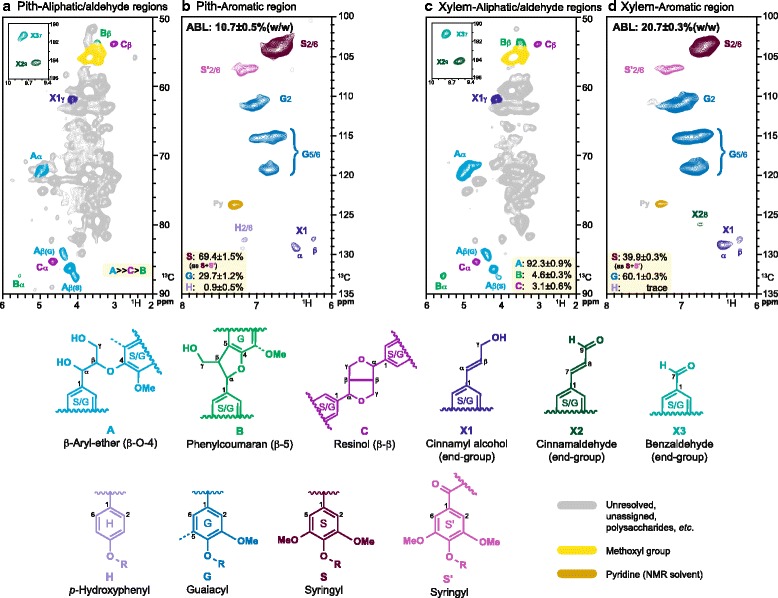
2D–HSQC-NMR spectra of pith and xylem tissues. **a**, **c** Aliphatic/aldehyde and **b**, **d** aromatic regions of ^1^H–^13^C (HSQC) spectra from cell wall gel samples in DMSO-d_6_:pyridine-d_5_ (4:1). **a**, **b**: Pith tissue. **c**, **d**: Xylem tissue. Levels of **A**, **B** and **C** in pith (A) were not quantified because of co-located polysaccharides with **A**α, however the spectra allow the relative estimation. NMR lignin correlations are coloured to match the structures responsible. ABL refers to total lignin content measured as acetyl bromide lignin

Samples were prepared for 2D–NMR for each seed fibre, pith, and xylem tissue [60]. Freeze dried tissues were cut into small pieces and pre-ground using a Mixer Mill MM 400 (Retsch) under the condition of 30 s^−1^ vibrational frequency for 30–180 s, depending on the amount of sample. The pre-ground samples were extracted successively under ultra-sonication with distilled water (20 min, three times), 80% aqueous ethanol (20 min, three times) and acetone (20 min, twice). After drying in a freeze dryer, samples were ball-milled using a Fritsch planetary micro mill Pulverisette 7 vibrating at 600 rpm with zirconium dioxide (ZrO_2_) vessels containing ZrO_2_ ball bearings (10 mm × 10). The ball-milled pith and xylem tissues were gelled in DMSO-d_6_/pyridine-d_5_ (4:1) for NMR analysis. To concentrate cell wall components other than polysaccharides, the ball-milled seed fibre was subjected to digestion (72 h × 2) using Cellulysin® cellulase, *Trichoderma viride* (Calbiochem), at 35 °C in acetate buffer (pH 5.0). The residues after the enzyme digestion were dissolved into DMSO-d_6_/pyridine-d_5_ (4:1). NMR spectra were acquired on a Bruker Biospin AVANCE 700-MHz spectrometer fitted with a cryogenically-cooled 5-mm quadruple-resonance ^1^H/^31^P/^13^C/^15^N QCI gradient probe with inverse geometry (proton coils closest to the sample). ^1^H–^13^C HSQC experiments were carried out using the standard Bruker pulse sequence ‘hsqcetgpsisp2.2’ (phase-sensitive gradient-edited 2D HSQC using adiabatic pulses for inversion and refocusing), using the following parameters: acquired from 11.5 to −0.5 ppm in F2 (^1^H) with 1682 data points (acquisition time 100 ms), from 215 to −5 ppm in F1 (^13^C) with 620 increments (F1 acquisition time 8.0 ms) with a 0.5 s interscan delay (D1); the d_24_ delay was set to 0.86 ms (1/8 J, J = 145 Hz). Processing used typical matched-Gaussian apodization (GB = 0.001, LB = −0.1) in F2, and squared cosine-bell and one level of linear prediction (32 coefficients) in F1. The central DMSO peak was used as internal reference (δ_C_: 39.51, δ_H_: 2.49 ppm). Volume integration of contours was performed on data reprocessed without linear prediction, and used Bruker’s TopSpin 3.2 (Mac) software. For S/G/H quantification, the S2/6 (C_2_-H_2_/C_6_-H_6_), G2 (C_2_-H_2_) and H2/6 (C_2_-H_2_/C_6_-H_6_) correlations were used, and the G2 integral was logically doubled. For measurements of major lignin interunit linkages in their β-O-4, β-β, and β-5 structures, the contours corresponding to **A**α, **B**α and **C**α (Fig. 1) were integrated.

### RNA extraction, cDNA synthesis, and quantitative real-time PCR (qPCR)

RNA was extracted from xylem, pith, and seed fibre samples using a Qiagen RNeasy plant miniKit following the manufacturer’s instructions, with the exception that the RLT buffer was modified to include dithiothreitol (0.31% *w*/*v*; Boehringer), proteinase K (0.33% *w*/*v*; Amresco K525), polyvinylpyrrolidone-40 (2% *w*/*v*; Sigma PVP-40) and that the RLT-incubation step was performed for approximately 5 min at 40 °C to increase RNA yield and quality as assessed on a NanoDrop spectrophotometer (Thermo Scientific). Rnase-free DNase (Qiagen) treatment was performed on-column as recommended. Quality of RNA intended for sequencing was confirmed on a BioAnalyzer 2100 (Agilent Inc.).

cDNA synthesis was performed on 700 ng RNA using Superscript III (Invitrogen) following the manufacturer’s instructions and an oligo(dT)_22_V primer. qPCR was performed with four technical replicates using diluted cDNA, specified primer pairs (Additional file 2), Fast SYBR Green Master Mix (LifeTechnologies), and a 7900HT Fast Real-Time PCR System (Applied Biosystems) using comparative quantitation analysis against an internal reference gene (Gh_D_13g1487).

### RNAseq

Individual sequencing libraries were prepared at the ACRF Biomolecular Resource Facility, The Australian National University, from total RNA from stem xylem and pith and seed fibre using in-house protocols. Each library had unique barcode adaptors and were pooled before sequencing across 5 lanes using Illumina HiSeq2000 according to the manufacturer’s protocol. All reads were aligned to the *G. raimondii* reference genome [61, 62] using TopHat [63] (version 2.0.9, default parameters). Aligned BAM files were further processed using Cufflinks [64] (version 2.1.1, default parameters) and Cuffmerge [64] was used to produce a non-redundant set of transcripts. Read counts were then generated for each gene in each sample using the intersectBed program [65] (BedTools version 2.16.2) and custom Perl scripts by using annotated gene locations. Unaligned reads were not further analysed.

Differential expression analysis was performed using edgeR [66] (version 2.6.12) between samples following the authors’ recommendations; read counts per gene were normalised using the trimmed-mean of M-values (TMM) method [67].

Gene set enrichment analysis was performed using G:Profiler [68, 69]. Benjamini-Hochberg [70] adjusted *p* value (false-discovery rate) thresholds of 0.05 were used to evaluate the overall number of differentially expressed genes and a *p*-value of <1e^−6^ to identify the more highly significant differentially regulated genes.

## Results and discussion

### Cotton plants have compositionally ‘atypical’ primary and secondary cell walls

We determined the cell wall composition, in particular the lignin and polysaccharide content, of stem xylem and pith (considered to be SCW and PCW tissue types, respectively) as well as seed fibres (a developmental series from PCW, transition to SCW and SCW deposition stages) to understand the variety of cell wall types present in cotton. Previous work had suggested that SCWs of mature seed fibres [54] and those of cotton stem pith [53] may be lignified, so we measured acetyl bromide lignin (ABL) in the cell walls from xylem, pith, and mature seed fibre and characterised the lignins by NMR (Fig. 1, Additional file 3). Stem xylem and pith from the same stem segments both contained high levels of lignin. Mature seed fibre, on the other hand, contained only low amounts of measurable ABL. NMR of mature and immature (25 DPA) seed fibre could not detect signals for guaiacyl nor syringyl lignin but could detect two peaks in aromatic regions that are consistent with the existence of *p*-hydroxphenyl units. If lignin does exist in cotton seed fibre it has an unusual composition in that it is not composed of the typical “G” and “S” subunits found in ‘normal’ dicot lignins. NMR indicated that the lignin in the pith tissue had a substantially higher S:G ratio (~70:30) than that in the xylem (~40:60). The higher S:G ratio of pith lignin was also reflected in its lignin structure as indicated by the larger peak of **A**β(S) than of **A**β(G) in the aliphatic region of the spectra.

To characterise polysaccharide composition, we performed monosaccharide linkage analysis on the cell walls from stem xylem, stem pith, and seed fibre at 7 DPA (PCW, SF07), at 14–16 DPA (transition to SCW, SF15), and at 25 DPA (SCW, SF25) (Table 1, Additional file 4). We found that although seed fibre walls at maturity were over 90% cellulose with almost no lignin, cell walls during fibre elongation (SF07) and the transition to SCW development (SF15) contained considerably less cellulose and had more pectic polysaccharides and the hemicelluloses xyloglucan, heteroxylan, and heteromannan than those fibres actively depositing SCWs at 25 DPA. The relative proportion of callose was very low in xylem and pith and low in 7 DPA walls, but was highest in the transition walls, as has also been reported by Maltby, Carpita [71]. The cotton stem xylem cell walls were more typical of other dicot SCWs being composed predominantly of cellulose, lignin, and heteroxylan. Stem pith cell walls, surprisingly, had a composition similar to that in xylem, but with a greater proportion of pectic polysaccharides with a higher degree of methyl esterification (DE). They also had relatively high levels of lignin, cellulose, and heteroxylan, all of which are not ‘typical’ of PCWs in Arabidopsis and some other plant species. It is therefore difficult to describe cotton stem pith as a classical PCW tissue, but considering that these cells lack any obvious secondary wall thickening and stain like primary walls with toluidine blue while giving a positive Maule reaction for lignin (Additional file 5), without further extensive analysis, we can only suggest that the pith in cotton is composed of cells with lignified PCWs and a composition that is ‘atypical’. Lignified PCWs have been described in dark-grown maize coleoptiles [72] and in cell suspension cultures of hybrid aspen [73].

**Table 1:** Cell wall composition of cotton stem xylem, stem pith, and seed fibres

	XYLM	PITH	SF07	SF15	SF25
Homogalacturonan	1.0 ± 0.1	4.0 ± 0.6	19.7 ± 5.0	13.7 ± 0.5	0.7 ± 0.4
Rhamnogalacturonan	0.6 ± 0.1	0.9 ± 0.02	4.3 ± 1.3	4.8 ± 0.2	0.4 ± 0.1
Arabinan	0.5 ± 0.2	0.7 ± 0.1	15.1 ± 1.8	8.6 ± 0.1	0.2 ± 0.1
Type I AG	0.3 ± 0.02	0.5 ± 0.1	2.9 ± 0.3	2.9 ± 0.1	0.1 ± 0.02
Type II AG	0.6 ± 0.1	0.4 ± 0.1	8.2 ± 0.2	8.2 ± 0.01	0.9 ± 0.01
Heteroxylan	27.6 ± 4.3	19.8 ± 7.1	2.9 ± 1.1	1.7 ± 0.1	0.6 ± 0.1
Callose	0.1 ± 0.001	0.1 ± 0.05	2.1 ± 0.5	12.2 ± 0.9	8.7 ± 2.5
Heteromannan	3.4 ± 0.5	2.8 ± 0.1	5.6 ± 0.3	5.6 ± 0.4	1.4 ± 0.02
Xyloglucan	4.8 ± 0.6	4.1 ± 0.7	9.5 ± 1.4	10.6 ± 0.7	1.4 ± 0.5
Cellulose	61.0 ± 5.9	66.7 ± 7.3	23.4 ± 0.1	29.2 ± 0.5	85.6 ± 3.4
Others	tr	tr	6.4 ± 0.2	2.6 ± 0.5	tr
4-GalA DE	42.5 ± 3.5	51.0 ± 5.7	49.0 ± 5.7	42.5 ± 3.5	20.0 ± 0.00

Polysaccharide composition of cell walls deduced from linkage analysis of cotton stem xylem (XYLM), stem pith (PITH), and 7, 15, and 25 DPA seed fibre (SF07, SF15, SF25) in mol%. Mean values ± SD are shown (*n* = 2). AG, arabinogalactan; 1,4-GalA DE, (1,4)-linked galacturonic acid degree of esterification, tr, <0.05 mol%

### Compositional differences between stem tissues and seed fibres are reflected in their tissue-specific transcriptomes

**Fig. 2:**
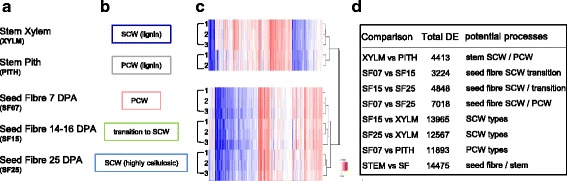
Summary of differentially expressed genes in cotton stem xylem, stem pith, and seed fibres. **a** Tissues sampled for transcriptome analysis. These included stem xylem (XYLM), stem pith (PITH), and seed fibre at 7 DPA (SF07), 14, 15, 16, DPA (SF15), and 25 DPA (SF25). DPA denotes Days Post Anthesis. **b** Typical cell walls found in each tissue type. **c** Hierarchical clustering of the differentially expressed genes across the stem and seed fibre samples. The biological replicate of each tissue is shown as a number (1–3). **d** Total overall number of differentially expressed genes in various comparisons between developmental or tissue types, at a *p*-value of 0.05. The processes that these differentially expressed genes might potentially be involved in is noted. SCW, secondary cell wall; PCW, primary cell wall

Even though they are both considered to be SCWs, the cell walls of mature cotton seed fibres and stem xylem are very different compositionally (Table 1). To determine which genes contribute to these different types of SCWs, we performed RNA sequencing on the five different cotton *(G. hirsutum)* tissues described earlier (Fig. 2a, b). Sixty-five percent of the sequencing reads mapped to the *G. raimondii* D-genome, enabling us to determine transcript identity and relative abundance. The identified genes were annotated based on the amino acid similarity of the encoded proteins to the closest corresponding Arabidopsis proteins and their annotations (TAIR10, [74]). We note that tissues and genes of *G. hirsutum* (A + D Genome), a highly spinnable cotton, were mined here by using the cotton reference genome publically available at the time, *G. raimondii* (D-genome); future studies using a reference genome of *G. hirsutum* could provide valuable insights. Hierarchical clustering confirmed that the three biological replicates of each tissue type (only two replicates in the case of the pith from which it was difficult to extract enough high quality RNA) clustered together (Fig. 2c). As found for the xylem and seed fibre replicates, the two pith replicates had very similar results. We found that the stem xylem and pith transcriptomes were more similar to each other than they were to those of the seed fibre and that the different seed fibre transcriptomes showed more similarity to each other than to either of the stem transcriptomes (Fig. 2c).

Differential expression comparisons were performed amongst the transcriptomes of the different tissues and cells to identify genes important for cotton SCW biogenesis (Fig. 2d). Approximately 12,500 genes were differentially expressed (at an adjusted *p*-value of 0.05) between the SF25 and stem xylem samples (Fig. 2d). Comparing SCW and PCW tissue types, approximately 7000 genes were differentially expressed between SF25 and SF07, and approximately 4400 genes between stem xylem and pith samples (Fig. 2d). Comparison of SF15 with SF07 or SF25 revealed about 3000 and 5000 genes differentially expressed at the transition to seed fibre SCW synthesis, respectively (Fig. 2d).

We examined expression levels (as FKPM – fragments per kilobase of million reads mapped) in more detail for the following differentially expressed gene classes: NACs, MYBs, WRKYs, auxin-related TFs, KNATs, BEL1-likes, bHLH TFs, cellulose synthase-related, phenylpropanoid pathway-related, and FLAs. Some but not all of these classes contain members that are considered key components of the current ‘typical’ SCW gene regulatory network.

### Homologs of top tier NAC transcriptional regulators of secondary cell wall deposition show tissue-specific expression in cotton

**Fig. 3:**
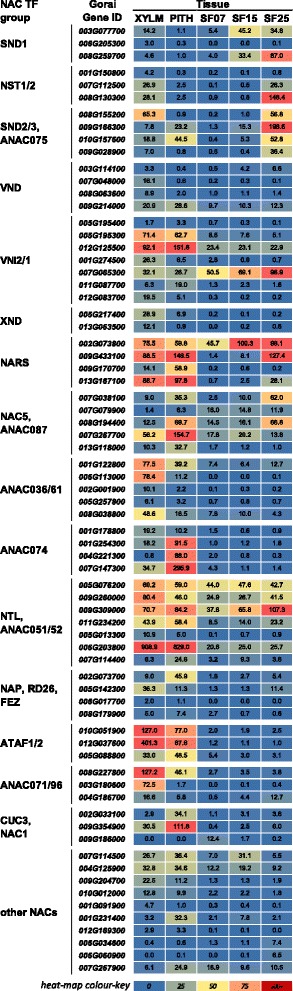
*NAC* transcription factor expression during *Gossypium hirsutum* SCW and PCW formation. The heat map shows the RNA expression level as normalised FPKM of each differentially expressed NAC across the five tissues sampled. The differentially expressed NAC transcription factors were classified into ‘NAC TF groups’ based on phylogenetic similarity with Arabidopsis NACs (Additional file 7). XYLM, stem xylem; PITH, stem pith; SF07, seed fibre at 7 DPA; SF15, seed fibre at 14, 15, 16, DPA; SF25, seed fibre at 25 DPA; DPA, days post anthesis

**Fig. 4:**
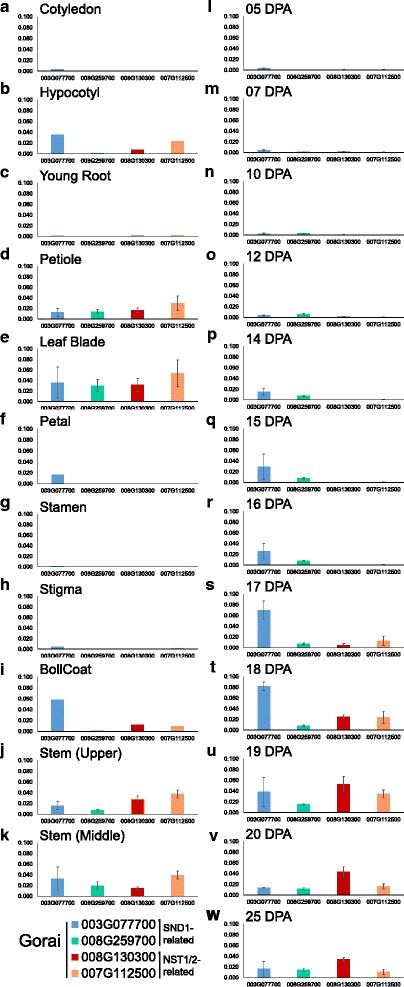
qPCR tissue profiling of select SND1 and NST1 NACs differentially expressed during cotton SCW development. qPCR based expression analysis was performed for key NACs that were differentially expressed between the different cotton SCW and PCW cell types. Comparative expression relative to ubiquitin is shown as relative abundance. These were SND1-like NACs (**a**) *Gorai.003G077700* and (**b**) *Gorai.008G259700*, as well as NST1-related NACs (**c**) *Gorai.007G112500* and (**d**) *Gorai.008G130300*. Expression profiling of the identified NACs in RNA from a range of tissues including **a** cotyledon, **b** hypocotyl, **c** young root, **d** petiole, **e** leaf blade, **f** petal, **g** stamen, **h** stigma, **i** boll coat, **j** upper stem, **k** middle stem, and a seed fibre developmental series from **l** 5 DPA, **m** 7DPA, **n** 10DPA, **o** 12 DPA, **p** 14 DPA, **q** 15 DPA, **r** 16 DPA, **s** 17 DPA, **t** 18 DPA, **u** 19 DPA, **v** 20 DPA, **w** 25 DPA Mean values ± SD are shown (*n* = 3), otherwise single replicates are shown. n.d., nil detected; DPA, days post anthesis

Several members of the NAC TF family are considered key regulators of Arabidopsis SCW biogenesis pathways [1–3]. In our data, we found 13 NAC groups that were differentially expressed between the different cotton samples (Fig. 3, Additional files 6 and 7). SND1- and NST1/2-related genes were expressed specifically in xylem cells and/or seed fibre with SCWs, in agreement with the current model for Arabidopsis which places SND1 and NST1/2 in a top tier. Notably, the two *SND1-*like genes with notable expression levels (*Gorai003G0777000* and *Gorai008G259700*) were already expressed at the transition stage, whereas the *NST1/2*-related genes were only expressed at the 25 DPA (SCW) stage. The earlier onset of *SND1*-*like*- compared to *NST1/2*-expression was confirmed using quantitative real-time PCR in an independent set of samples including additional seed fibre stages and other plant tissues (Fig. 4). Tuttle JR, et al. [55] also found *Gorai003G0777000-* and *Gorai008G259700-*homologous genes in *G. barbadense* and *G. hirsutum* seed fibre to be up-regulated at 15 DPA compared to 10 DPA. Although they concluded that these are NST1-related NACs, the phylogenetic analyses from this study indicate that these genes are more related to the SND1 types (Additional file 7); based on the known literature and nucleotide and amino acid phylogenetic analyses, the currently best estimation is that they are SND1-*like*. SND1-*like* NACs therefore appear to act earlier than NST1/2 in cotton seed fibre SCW development unlike in Arabidopsis where SND1 and NST1/2 are thought to be equivalent top tier TFs. The Arabidopsis stem xylem system is not conducive to transcriptome analysis at the transition stage, so it may be possible that SND1 acts, at least temporally, upstream of NST1 in Arabidopsis and other plant species. In support of this, transformation of Arabidopsis with ProSND1:GUS resulted in GUS expression in xylem fibre during elongation prior to SCW deposition [11] while this may not be the case for ProNST1:GUS [15].

In Arabidopsis the second or middle tier NAC, SND2, is considered to act downstream of SND1/NST1 in the xylem fibre SCW network [8, 21] and this appears to be similar in cotton seed fibre SCW development. The cotton *SND2/3*-related transcripts were observed in 25 DPA, but not in 7 DPA or 15 DPA seed fibre, whereas *SND1-like* expression was already induced in 15 DPA seed fibre (Fig. 3). One of those *SND2-*related NAC genes, *Gorai009G166300*, showed predominant expression in 25 DPA fibres and had the highest transcript level of any of the NAC-related genes across the seed fibre series. In Arabidopsis, SND2 is able to directly activate the *CesA8* promoter [8] and some have suggested it specifically controls cellulose, mannan, and xylan biosynthesis, as well as lignin polymerization, but not monolignol synthesis [75], and thus *Gorai009G166300* may have a similar role in cotton fibres (excluding lignin polymerization as that does not occur in the fibre SCW).

In Arabidopsis, VND-related TFs regulate SCW deposition in xylem vessels and are considered to be top tier TFs together with SND1 and NST1/2 [8, 9]. In our samples, *VND*-related genes showed expression mainly in xylem, but not much in seed fibre (Fig. 3). SND1- and NST1/2-related TFs are therefore likely to be top tier SCW NACs in seed fibre SCW biogenesis, whereas they may be accompanied by VND-related TFs in cotton stem xylem.

There was no consistent expression pattern for the *VNI2/1*-related genes in our samples (Fig. 3). However, two of the genes (*Gorai005G195300* and *Gorai012G125500*) showed highest expression in xylem and pith, indicating that they have a specific function in stem. In contrast, *Gorai007G065300* showed increasing transcript levels in seed fibre from 7 to 25 DPA, with lower expression in the stem samples. It has been suggested that VNIs can function as repressors of NAC function by interacting with other NAC TFs [18] and this may also be the case in cotton seed fibre.

In Arabidopsis and poplar, XND1 is thought to act as a repressor of xylem SCWs [76]. In our cotton samples we observed moderate levels of expression of *XND1*-related genes in xylem, but they were completely absent from seed fibre (Fig. 3).

Members of several NAC groups, that have not previously featured in SCW regulation, were specifically up-regulated to sometimes high levels in cotton xylem compared to pith or seed fibres, and these include ANAC036/61- (*Gorai001G122800, 006G113000*), ANAC071/96- (*Gorai003G180600, 008G227800*) and ATAF1/2-related NACs (*Gorai012G037600*) (Fig. 3). These NACs are good candidates for future studies of cotton xylem SCW development. We also found some NAC-related TFs that were expressed highly in just pith compared to xylem or seed fibres, i.e., ANAC074 (*Gorai001G254300, Gorai004G221300, Gorai007G147300*) and CUC3/NAC1-related (*Gorai009G354900*) NACs. In contrast, few of the identified NAC-related genes showed particularly high expression in elongating or transition stage seed fibres. This indicates that NAC TFs, with the exception of the SND1-related genes, do not play a major role at those developmental stages.

Our data are in general agreement with the current model of SCW regulation in Arabidopsis as SND1-, NST1/2-, and VND-related NACs are all present in cotton cells undergoing SCW deposition and are amongst the early genes to be induced. However, a further level of detail might have to be added to the current model as our seed fibre developmental series strongly suggests that SND1-like TFs act earlier than NST1/2-related TFs in the SCW regulatory network. Additionally, many so far uncharacterised members of the NAC TF family displayed xylem-specific expression, suggesting possible roles for these genes in the lignocellulosic SCW gene regulatory network different to the highly cellulosic network found in seed fibre and these warrant further investigation.

### Important ‘MYB master switches’ are missing in some cotton tissues with secondary cell walls

**Fig. 5:**
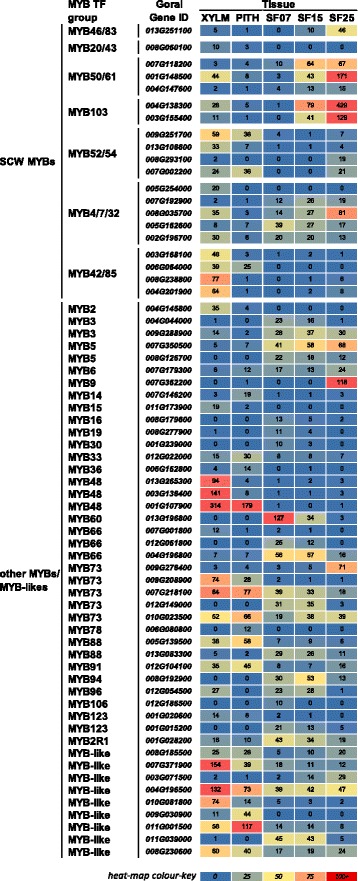
*MYB* transcription factor expression during *Gossypium hirsutum* SCW and PCW formation. The heat map shows the RNA expression level as normalised FPKM of each differentially expressed MYB across the five tissues sampled. The groups are MYBs related to Arabidopsis SCW MYBs, or other MYBs/MYB-like genes. XYLM, stem xylem; PITH, stem pith; SF07, seed fibre at 7 DPA; SF15, seed fibre at 14, 15, 16, DPA; SF25, seed fibre at 25 DPA; DPA, days post anthesis

Several MYB TFs have been described as being important for SCW deposition or composition [19–23]. We identified 65 *MYB* or *MYB-like* genes that were differentially expressed in at least one of the analysed comparisons (Fig. 5, Additional file 6). Twenty of these were putative homologs of MYB TFs known to be involved in regulation of SCW or lignin synthesis in Arabidopsis. Amongst these MYB genes, those encoding putative MYB50/61 and MYB103 homologs showed very high transcript levels in 25 DPA seed fibres, but low levels in xylem. Expression of these MYBs began to be induced in 15 DPA seed fibre and continued to increase until 25 DPA. AtMYB50/61 have yet to be confirmed to have a role in SCW development, but our results would support such a function in cotton seed fibre. AtMYB103, on the other hand, has been shown to regulate expression of *AtF5H1* and consequently the S:G ratio of lignin in Arabidopsis [77]. Additionally, AtMYB103 has been identified as a direct target of the top tier NAC TFs and as a strong activator of the *AtCesA8* promoter [8]. Induction of the two *MYB103*-related genes in cotton seed fibre correlated well with the induction of the *SND1*-*like* genes in the same samples so they may also positively regulate MYB103 expression in seed fibre. The possible downstream targets of MYB103 TFs in cotton will be discussed later, but it is worth mentioning that both *F5H1*- and *CesA8*-related gene expression correlated well with the temporal and tissue-specific expression of the two *MYB103*-related genes.

Transcripts of genes encoding putative *MYB42/85* homologs were almost exclusively present in xylem, in agreement with their proposed function in Arabidopsis in activating transcription of genes in the lignin-specific part of the phenylpropanoid pathway [8]. The absence of notable levels of *MYB42/85*-related gene transcripts and presumably also protein in cotton seed fibre provides at least a partial explanation for the low lignin content observed for these cells. One of the *MYB42/85*-related genes (*Gorai006G064000*) was expressed at moderate levels in pith. However, whether this is the reason for the surprisingly high lignin content of cotton pith cell walls requires further analysis. AtMYB58/63 have also been found to positively regulate lignin deposition in Arabidopsis [78]. However, the expression levels of two putative MYB58/63-related genes (*Gorai004G269400* and *Gorai002G106300*) were almost negligible in the lignin-containing xylem and absent from the other samples, including pith, indicating that MYB58/63 homologs do not play a major role in lignification of cell walls in cotton. We did identify five genes encoding putative homologs of AtMYB4/7/32, known repressors of lignin biosynthetic genes [46–48], that had moderate transcript levels in xylem and/or seed fibre, but no notable amounts in pith. The relatively high transcript level of the *AtMYB4/7/32*-related *Gorai008G35700* in 25 DPA fibre could be another explanation for the absence of notable lignin deposition in seed fibres. On the other hand, transcript levels of *MYB4/7/32*-related genes in pith and xylem were not negatively correlated with the lignin level in the walls of those cells, which argues for a different function for these MYBs in cotton stems.

We identified only one gene with detectable expression that potentially encodes a MYB46/83 homolog (*Gorai013G251100*), with its transcript mainly present in the 25 DPA sample. No notable amounts of transcript were detected for any of the other genes that potentially encode MYB46/83 homologs (*Gorai006G129100*, *Gorai001G138800*, *Gorai004G172700*, *Gorai004G037300*). Arabidopsis AtMYB46/83 and homologs in several other plant species can activate the entire suite of SCW biosynthetic programs [79]. The very low level of expression in cotton stem xylem with its more ‘typical’ SCW, suggests that there are ways of regulating composition of these cell walls other than those in Arabidopsis.

For many of the genes in the group we have classed as “other MYBs” we found tissue-specific expression patterns, suggesting specific functions in different cell types. The three genes encoding putative AtMYB48 homologs, for example, had the highest transcript levels for any MYBs across all samples. However, their expression was entirely absent from seed fibres, while only one of the genes was expressed in pith (*Gorai001G107900*). To our knowledge, AtMYB48 has not been previously associated with SCW formation, but our results strongly suggest at least a potential xylem-specific function for the cotton homologs.

Another player in the current ‘typical’ SCW regulatory network that is apparently missing in cotton is MYB75. Arabidopsis AtMYB75 is a positive regulator of anthocyanin synthesis and together with AtKNAT7 represses SCW synthesis [80]. The cotton genome does not appear to encode a protein with significant similarity to AtMYB75, but it does encode at least 3 KNAT7 homologs (Additional file 8, C) two of which have their highest expression in 25 DPA seed fibres.

### WRKY transcription factors are involved in stem, but not seed fibre SCW development

**Fig. 6:**
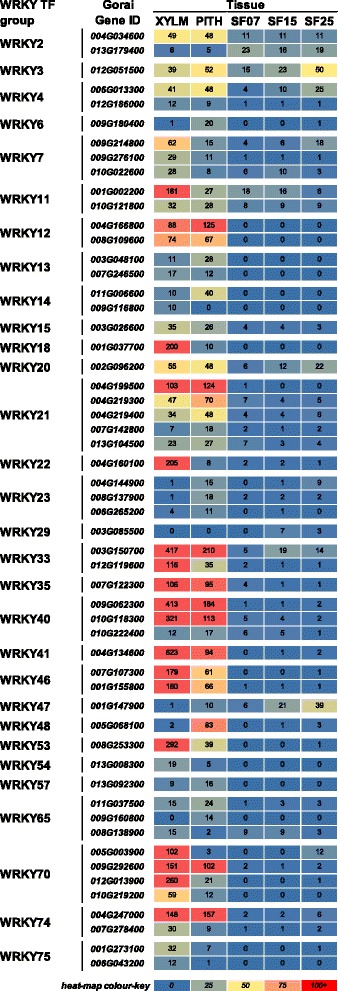
*WRKY* transcription factor expression during *Gossypium hirsutum* SCW and PCW formation. The heat map shows the RNA expression level as normalised FPKM of each differentially expressed WRKY across the five tissues sampled. XYLM, stem xylem; PITH, stem pith; SF07, seed fibre at 7 DPA; SF15, seed fibre at 14, 15, 16, DPA; SF25, seed fibre at 25 DPA; DPA, days post anthesis

We identified 55 *WRKY* genes that were differentially expressed in at least one of the analysed comparisons (Fig. 6, Additional file 6). In xylem and pith, most of the *WRKY* genes were expressed at comparable levels or had a predominance in one of the two tissue types. Only three genes showed similar or higher transcript levels in seed fibre compared to xylem and/or pith, a *WRKY2* (*Gorai013G179400*), a *WRKY3* (*Gorai012G051500*), and a *WRKY47* (*Gorai001G147900*) homolog, but expression of these was generally low compared to other members of the family in xylem/pith. AtWRKY12 and its homolog in Medicago are the only WRKY TFs that have been shown to be involved in SCW regulation, where they repress AtNST2 and loss of function of AtWRKY12 in Arabidopsis results in ectopic lignin, xylan, and cellulose deposition in stem pith [81]. Transcripts of genes encoding putative cotton *WRKY12* homologs were only detected in xylem and pith, but not in seed fibre. Strikingly, in seed fibre, WRKY TFs are generally low or not expressed at all. Ding, Chen [82] reported that WRKY TFs may play a role in seed fibre development, based on their differential expression at different times relative to 0 DPA in *G. arboreum* and *G. raimondii*. Our data in *G. hirsutum*, however, suggests that WRKY TFs may be more important in stem tissues than in seed fibre development.

Other classes of TFs with differentially expressed genes across the comparisons were auxin/IAA-related, BEL1-like, and bHLH TFs (Additional files 6 and 8). We identified 21 auxin/IAA-related TF genes (Additional file 8, A), with many of them having high transcript levels in xylem and/or pith. Two genes, one *IAA14/SLR*- and one *IAA30*-related gene (*Gorai006G246000* and *Gorai007G117700*, respectively) showed seed fibre-specific expression: *Gorai006G246000* showed moderate expression at 7 DPA and highest transcript level at 15 DPA, whereas *Gorai007G117700* was exclusively expressed at 15 DPA. These two TFs are potential candidates as activators of SCW synthesis at the seed fibre transition stage, but further experiments are required to test their function. In Arabidopsis, IAA14/SLR has been shown to negatively regulate lateral root initiation [80] and IAA30 has been shown to be specifically expressed in the quiescent centre of the root apical meristem [83], but their specific mode of action is still unclear. BEL1-like TFs were highly expressed in xylem and pith, but with no clear predominance in either tissue and some of the genes were also expressed in seed fibre, especially at 25 DPA (Additional file 8, B). Consequently, there is no clear bias in expression of these TFs in cells with PCW or SCWs. bHLH-related genes had very little expression in seed fibre compared to stem (Additional file 8, D). Two exceptions were *Gorai002G016500* and *Gorai009G176000* which were much more highly expressed than in xylem suggesting a seed fibre SCW-specific role. Cotton xylem had two bHLH genes, *Gorai011G292100* and *Gorai013G242800*, with high levels compared to any of the other samples, suggesting that these may play a specific role in lignocellulosic SCW synthesis.

### The cotton seed fibre is a ‘cellulose synthesis machine’

**Fig. 7:**
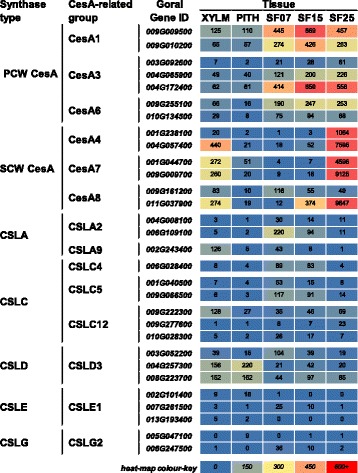
*CesA* and *CSL* expression during *Gossypium hirsutum* SCW and PCW formation. The heat map shows the RNA expression level as normalised FPKM of each differentially expressed PCW CesA, SCW CesA, and CSL-like gene across the five tissues sampled. XYLM, stem xylem; PITH, stem pith; SF07, seed fibre at 7 DPA; SF15, seed fibre at 14, 15, 16, DPA; SF25, seed fibre at 25 DPA; DPA, days post anthesis

Specific sets of CesAs are known to be involved in synthesizing SCWs (or PCWs). We determined which *CesA* genes were expressed in the different tissues in cotton (Fig. 7, Additional file 6; the CesA groupings were assigned based on their homology to Arabidopsis genes). The PCW CesAs (*CesA1*-, *CesA3*- and *CesA6*-related) were expressed relatively highly across the seed fibre developmental stages, including SF25, with lower abundance in xylem and pith. In contrast, SCW *CesA* transcript levels were very high in SF25, with the exception of one *CesA8*-related gene (*Gorai011G037900*), and were hardly expressed in the other two seed fibre samples or pith. Expression levels of most of the genes in xylem were relatively high, but still notably lower than in 25 DPA fibre. *Gorai011G037900* was the only SCW *CesA*-related gene that was already expressed in 15 DPA seed fibre and continued to increase up to 25 DPA. This pattern correlates well with the expression of the two *MYB103*-related genes we identified (Fig. 5), suggesting that, as in Arabidopsis where they strongly activate the At*CesA8* promoter [8], these MYBs may regulate specific CesA expression in cotton. One of the *CesA4* genes, *Gorai001G238100*, was specific to the seed fibre SCW stage, with high expression at 25 DPA and very low expression in xylem tissue. *Gorai009G161200*, another putative SCW *CesA*-related gene, was found to be most highly expressed in 7 DPA seed fibre, but its expression levels were generally low compared to the expression levels of other PCW and SCW *CesA*-related genes. In conclusion, the expression patterns for the various classes of *CesA*-genes across the samples were in general agreement with their respective classification as either PCW or SCW CesAs [31]. Furthermore, the much higher expression levels of SCW CesAs at 25 DPA in seed fibre compared to xylem is indicative of the seed fibre’s ultimate dedication to produce cellulose and is in agreement with previous findings [55].

The cellulose synthase-like genes (*CSLs*) identified in this study include *CSLA*, *CSLC*, *CSLD*, *CSLE*, and *CSLG*. In contrast to the *CesA*s, the *CSL* transcripts were generally low in abundance in all samples. AtCSLA2 and AtCSLA9 reportedly act in the synthesis of glucomannan in Arabidopsis stems [84], but it seems that amongst the homologs detected here, at least one *CSLA2* gene is early seed fibre-specific. The *CSLA9* homologs were most highly expressed in stem xylem, consistent with their known role in Arabidopsis stems. There is evidence for the involvement of *CSLCs* in xylogucan biosynthesis [85] and we saw expression of CSLC homologs in 7 and 15 DPA seed fibre and the stem xylem samples, consistent with the presence of xyloglucans in these tissues (Table 1). CSLDs, including CSLD3, have been suggested to be involved in mannan biosynthesis [86]. We saw expression of *CSLD3*-related genes at reasonably high levels in stem tissue, but lower levels in fibre, except for *Gorai003G052200*, which was high-moderately expressed across the tissue samples examined.

### Several FLAs are likely to be key proteins in cell wall deposition and function in seed fibre

**Fig. 8:**
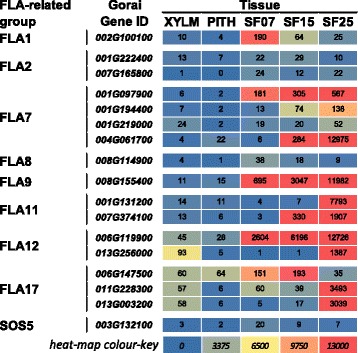
*FLA* expression during *Gossypium hirsutum* SCW and PCW formation. The heat map shows the RNA expression level as normalised FPKM of each differentially expressed FLA gene. XYLM, stem xylem; PITH, stem pith; SF07, seed fibre at 7 DPA; SF15, seed fibre at 14, 15, 16, DPA; SF25, seed fibre at 25 DPA; DPA, days post anthesis

A small sub-group of FLAs has been implicated in SCW structure and function [37], so we examined which cotton *FLAs* were expressed in tissues depositing SCWs. Of the 17 differentially expressed *FLA*s, 11 had seed fibre-specific expression (Fig. 8, Additional file 6). Additionally, expression of nine of the genes was extremely high at 25 DPA, including *FLA7*-, *FLA9*-, *FLA11*-, *FLA12*-, and *FLA17*-related genes. FLA proteins have previously been reported as some of the most abundant glycoproteins in developing seed fibre [87]. In contrast to the wide range of various FLA genes expressed in cotton seed fibre, only *FLA12-* and *FLA17*-related genes showed any notable expression in xylem. These expression patterns of *FLA* genes are strongly indicative of FLA proteins being key components of cotton seed fibre cell walls, some especially specific to the SCW synthesis stage.

Several other genes associated with cellulose synthesis, structure, and deposition were also very highly expressed in 25 DPA seed fibre, including: *COBL4*-related (*Gorai004G063600*, *Gorai007G176400*), *CTL2*-related (*Gorai006G078900*, *Gorai011G198500*), *FRA1* (*Gorai002G1*34500), *HERK1* (*Gorai001G107000*), and *KOR1* (*Gorai010G143300*) (Additional file 9). Additionally, some putative xylan glycosyl transferase genes, such as *IRX9*-related (*Gorai006G168500*) and *IRX10*-related (*Gorai005G197500*), were also highly and specifically expressed in 25 DPA seed fibre.

### Phenylpropanoid pathway genes are highly expressed in tissues with lignified SCWs

**Fig. 9:**
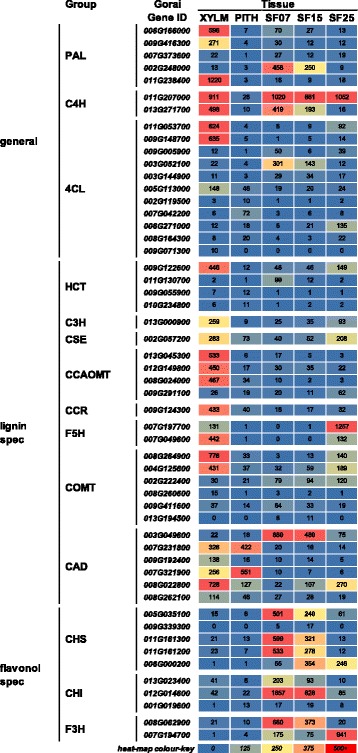
Phenylpropanoid pathway gene expression during *Gossypium hirsutum* SCW and PCW formation. The heat map shows the RNA expression level as normalised FPKM of each differentially expressed phenylpropanoid pathway gene. These are grouped as general, lignin- specific, and flavonol-specific phenylpropanoid pathway genes. Spec, specific; XYLM, stem xylem; PITH, stem pith; SF07, seed fibre at 7 DPA; SF15, seed fibre at 14, 15, 16, DPA; SF25, seed fibre at 25 DPA; DPA, days post anthesis

We identified 43 lignin-specific or general phenylpropanoid pathway genes that were differentially expressed in at least one of the analysed comparisons (Fig. 9, Additional file 6). Additionally, ten genes encoding the first three enzymes of the flavonoid-specific pathway, an early branch of the general phenylpropanoid pathway, were also differentially expressed. Expression of general and lignin-specific pathway genes in xylem and pith, tended to be more xylem-specific (low in pith), with exception of members of the CAD family that showed comparable transcript levels in both tissues. Additionally, most phenylpropanoid genes and especially genes of the lignin-specific pathway were lowly or not expressed in seed fibre compared to xylem, consistent with the absence of the known activators of the lignin pathway, such as the MYB42/85-related TFs, in these cells. The low abundance of lignin-specific transcripts in seed fibre has been reported previously [55] and provides an explanation for the lack of significant lignification of seed fibre SCWs. However, the absence of lignin-specific transcripts (with the exception of *CAD*) in pith was somewhat unexpected, as we observed a relatively high lignin content for the cell walls of these cells (Fig. 1). It is possible that although the cells of the pith do not participate in making monolignols themselves, they can convert the aldehydes that are either actively or passively transported to them from surrounding monolignol-making cells, to their respective alcohols via CAD activity and complete the lignification process. There is indeed evidence that monolignols can be transferred to other cells/tissues to be polymerised into lignin [41].

Curiously, the highest transcript level for one of the genes encoding a *F5H* homolog (*Gorai.007G197700*) was in 25 DPA seed fibre with no notable expression in any of the other seed fibre samples or pith and approximately ten times less in xylem, despite there being no substantial monolignol synthesis in this tissue. As mentioned earlier, F5H expression is directly activated by MYB103 in Arabidopsis [77]. However, MYB103 has also been shown to regulate CesA8 expression [8], a regulatory step that most likely contributes to efficient cellulose synthesis in SF25 as discussed earlier. Similarly, we detected high levels for one *CAD*-related gene (*Gorai003G049600*) specifically in 7 and 15 DPA seed fibre. The roles of these genes in this tissues are unknown, but it is possible that both F5H and CAD enzymes, if produced, are used in metabolic pathways, such as in secondary metabolite or cuticle formation, rather than in monolignol production.

In contrast to the genes encoding enzymes of the lignin-specific phenylpropanoid pathway, there was seed fibre-specific expression of genes encoding enzymes of the flavonoid-specific pathway, with the highest transcript levels in elongating fibres at 7 and 15 DPA. We also identified at least one gene encoding each of the enzymes of the general pathway (PAL, C4H, and 4CL) that showed high transcript levels in at least one of the seed fibre samples. This suggests that these enzymes are providing the precursors for flavonol rather than for monolignol synthesis in seed fibre. Seed fibres indeed contain flavonoids and expression of flavonoid synthesis-related genes in seed fibre has been reported previously [55, 88]. The function of flavonoids in seed fibre has not been established, but it is generally accepted that flavonoids have various functions in plants including responses to abiotic and biotic stress. Suppressed development of seed fibre upon repression of *F3H* in cotton indicates, however, that flavonoids are necessary for seed fibre development [89].

## Conclusions

Despite both having SCWs, cotton seed fibres and xylem tissues are very different in both cell wall composition and transcript profiles. Cotton seed fibre transcriptomes after about 15 DPA show that they become dedicated to the production of their main cell wall polysaccharide, cellulose, leading to their unusual highly cellulosic SCWs that are essentially devoid of any lignin at maturity. It has been suggested [55, 56] that SCW production during seed fibre development has been re-programmed during domestication and selection in modern breeding to suppress lignin biosynthesis whilst recruiting stress-response genes needed to achieve greater fibre cell length and make cotton fibres more useful for textiles, and this is evident in our transcriptome data.

We used stem pith as an example of a non-seed fibre PCW tissue, but discovered that cotton pith walls appear to be another example of an ‘atypical’ dicot wall. Histology and transcript analysis suggest they are PCWs, but compositional analysis detected significant levels of lignin and xylan normally characteristic of SCWs. Few studies have compared the chemistry of PCWs and SCWs from the same species and in the same part of the plant, so it is difficult to determine if this truly is unusual or specific to cotton.

Comparisons between different tissues within cotton have indicated that cotton SCW deposition, like that in Arabidopsis, is probably regulated by a hierarchical cascade of transcriptional activation and repression that regulates cell wall polysaccharide, lignin, and protein composition. Top tier factors like SND1/NST1 and VND, that are conserved between cotton and Arabidopsis, initiate cotton SCW development, but key MYB regulators in lower tiers are missing from seed fibres and in the other tissues are different to those prominent in Arabidopsis, explaining their ‘atypical’ cell wall compositions. Even stem xylem that contains a more ‘typical’ SCW, has a regulatory network that is different in detail to those described for other species.

**Fig. 10:**
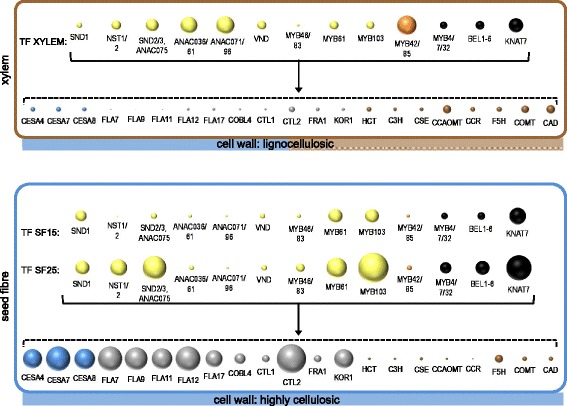
Concept map of cotton TF and cell wall genes for SCW synthesis. TF and cell wall gene expression in stem xylem (lignocellulosic cell walls), and in seed fibres (highly cellulosic cell walls) at transition to (SF15) and during (SF25) SCW synthesis. Bubble area is relative to transcript abundance of all the transcripts for each named gene/class. For visualisation, TF bubble size is at a larger scale (80×) than the biosynthetic and structural genes. Colour key: *yellow* - TF activators, *orange* – lignin-specific TF activator, *black* - TF repressors, *blue* - CesAs, *grey* - cellulose synthesis, structure and wall integrity, bronze - monolignol biosynthetic genes

Figure 10 summarises our key findings on cotton SCW biogenesis in both seed fibre and xylem. In xylem SCW development, a number of key NAC TFs, including ANAC036/61, ANAC071/96, VND, SND2/3, NST1/2, and the MYB TF MYB61 are some of the most abundant of the SCW activators. The lignin transcriptional activator MYB42/85 is also very abundant. In this tissue, the monolignol biosynthetic genes are far more abundant than those for cellulose synthesis and deposition or for cell wall structural proteins. The situation in seed fibres, however, is quite different. At the transition to SCW deposition, SND1*-like*, MYB103, and MYB61 are amongst the most abundant transcriptional activators, while the repressors KNAT7 and MYB4/7/32 are also abundant. There is very little expression of the lignin transcriptional activator MYB42/85. By 25 DPA, there is an increased abundance of SND2, MYB103, and KNAT7, and to a lesser extent MYB61, SND1*-like*, NST1/2, while MYB42/85 expression remains low. Also at this stage there is very high expression of cellulose biosynthetic genes, and cell wall structural genes like FLAs. In contrast, the monolignol biosynthetic genes, except for *F5H*, are very low in abundance. SND1*-like* appears to act upstream of the other factors and together with MYB103, SND2, MYB61, and NST1/2 leads to the strong activation of cellulose production and some cell wall structural proteins, while MYB42/85 expression is repressed or not activated, resulting in the absence of lignin in theses ‘atypical’ SCWs that make cotton fibres so ideal for industrial uses.

## Additional files

Additional file 1: Cotton stem pith and xylem used in RNA extraction for deep-sequencing. (PDF 193 kb)

Additional file 2: Primer sequences for qPCR. (PDF 80 kb)

Additional file 3: Partial HSQC spectra of cotton seed fibres, along with those from a synthetic lignin and *Arabidopsis thaliana*. (PDF 134 kb)

Additional file 4: Monosaccharide linkage composition of cell walls. (PDF 153 kb)

Additional file 5: Cotton stem cell cross-sections stained with toluidine blue and Maule reaction. (PDF 361 kb)

Additional file 6: Differentially expressed transcription factor and structural genes. (XLSX 276 kb)

Additional file 7: Phylogenetic tree of NAC TFs differentially expressed during cotton SCW development. (PDF 1214 kb)

Additional file 8: Other TFs expressed during *Gossypium hirsutum* SCW and PCW formation. (PDF 30 kb)

Additional file 9: Other cell wall related genes that were differentially expressed across the cotton tissues. (PDF 17 kb)

## References

[1] Hussey SG, Mizrachi E, Creux NM, Myburg AA (2013). Navigating the transcriptional roadmap regulating plant secondary cell wall deposition. Front Plant Sci.

[2] Zhong R, Ye Z-H (2015). Secondary cell walls: biosynthesis, patterned deposition and transcriptional regulation. Plant Cell Physiol.

[3] Taylor-Teeples M, Lin L, de Lucas M, Turco G, Toal TW, Gaudinier A, et al. An Arabidopsis gene regulatory network for secondary cell wall synthesis. Nature. 2015;517:571–5. 10.1038/nature14099 PMC4333722 25533953

[4] Zhong R, McCarthy RL, Lee C, Ye Z-H (2011). Dissection of the transcriptional program regulating secondary wall biosynthesis during wood formation in poplar. Plant Physiol.

[5] Zhong R, Lee C, McCarthy RL, Reeves CK, Jones EG, Ye Z-H (2011). Transcriptional activation of secondary wall biosynthesis by rice and maize NAC and MYB transcription factors. Plant Cell Physiol.

[6] Handakumbura PP, Hazen SP (2012). Transcriptional regulation of grass secondary cell wall biosynthesis: playing catch-up with *Arabidopsis thaliana*. Front Plant Sci.

[7] Duval I, Lachance D, Giguère I, Bomal C, Morency MJ, Pelletier G, et al. Large-scale screening of transcription factor-promoter interactions in spruce reveals a transcriptional network involved in vascular development. J Exp Bot. 2014;65:2319–33. 10.1093/jxb/eru116 PMC4036505 24713992

[8] Zhong R, Lee C, Zhou J, McCarthy RL, Ye Z-H (2008). A battery of transcription factors involved in the regulation of secondary cell wall biosynthesis in Arabidopsis. Plant Cell.

[9] Yamaguchi M, Demura T (2010). Transcriptional regulation of secondary wall formation controlled by NAC domain proteins. Plant Biotechnol.

[10] Demura T, Tashiro G, Horiguchi G, Kishimoto N, Kubo M, Matsuoka N, et al. Visualization by comprehensive microarray analysis of gene expression programs during transdifferentiation of mesophyll cells into xylem cells. Proc Natl Acad Sci U S A. 2002;99:15794–9. 10.1073/pnas.232590499 PMC137795 12438691

[11] Zhong R, Demura T, Ye Z-H (2006). SND1, a NAC domain transcription factor, is a key regulator of secondary wall synthesis in fibers of Arabidopsis. Plant Cell.

[12] Zhong RQ, Richardson EA, Ye ZH (2007). Two NAC domain transcription factors, SND1 and NST1, function redundantly in regulation of secondary wall synthesis in fibers of Arabidopsis. Planta.

[13] Mitsuda N, Iwase A, Yamamoto H, Yoshida M, Seki M, Shinozaki K, et al. NAC transcription factors, NST1 and NST3, are key regulators of the formation of secondary walls in woody tissues of Arabidopsis. Plant Cell. 2007;19:270–80. 10.1105/tpc.106.047043 PMC1820955 17237351

[14] Mitsuda N, Ohme-Takagi M (2008). NAC transcription factors NST1 and NST3 regulate pod shattering in a partially redundant manner by promoting secondary wall formation after the establishment of tissue identity. Plant J.

[15] Mitsuda N, Seki M, Shinozaki K, Ohme-Takagi M (2005). The NAC transcription factors NST1 and NST2 of Arabidopsis regulate secondary wall thickenings and are required for anther dehiscence. Plant Cell.

[16] Zhong R, Lee C, Ye Z-H (2010). Functional characterization of poplar wood-associated NAC domain transcription factors. Plant Physiol.

[17] Xu B, Ohtani M, Yamaguchi M, Toyooka K, Wakazaki M, Sato M, et al. Contribution of NAC transcription factors to plant adaptation to land. Science. 2014;343:1505–8. 10.1126/science.1248417 24652936

[18] Yamaguchi M, Ohtani M, Mitsuda N, Kubo M, Ohme-Takagi M, Fukuda H, et al. VND-INTERACTING2, a NAC domain transcription factor, negatively regulates xylem vessel formation in Arabidopsis. Plant Cell. 2010;22:1249–63. 10.1105/tpc.108.064048 PMC2879754 20388856

[19] McCarthy RL, Zhong R, Ye Z-H (2009). MYB83 is a direct target of SND1 and acts redundantly with MYB46 in the regulation of secondary cell wall biosynthesis in Arabidopsis. Plant Cell Physiol.

[20] Zhong R, Richardson EA, Ye Z-H (2007). The MYB46 transcription factor is a direct target of SND1 and regulates secondary wall biosynthesis in Arabidopsis. Plant Cell.

[21] Ko J-H, Kim W-C, Han K-H (2009). Ectopic expression of MYB46 identifies transcriptional regulatory genes involved in secondary wall biosynthesis in Arabidopsis. Plant J.

[22] Patzlaff A, McInnis S, Courtenay A, Surman C, Newman LJ, Smith C, et al. Characterisation of a pine MYB that regulates lignification. Plant J. 2003;36:743–54. 10.1046/j.1365-313x.2003.01916.x 14675440

[23] Goicoechea M, Lacombe E, Legay S, Mihaljevic S, Rech P, Jauneau A, et al. EgMYB2, a new transcriptional activator from Eucalyptus xylem, regulates secondary cell wall formation and lignin biosynthesis. Plant J. 2005;43:553–67. 10.1111/j.1365-313X.2005.02480.x 16098109

[24] Zhong R, Lee C, Ye Z-H (2010). Global analysis of direct targets of secondary wall NAC master switches in Arabidopsis. Mol Plant.

[25] Persson S, Wei HR, Milne J, Page GP, Somerville CR (2005). Identification of genes required for cellulose synthesis by regression analysis of public microarray data sets. Proc Natl Acad Sci U S A.

[26] Brown DM, Zeef LAH, Ellis J, Goodacre R, Turner SR (2005). Identification of novel genes in Arabidopsis involved in secondary cell wall formation using expression profiling and reverse genetics. Plant Cell.

[27] Mellerowicz EJ, Sundberg B (2008). Wood cell walls: biosynthesis, developmental dynamics and their implications for wood properties. Curr Opin Plant Biol.

[28] McFarlane HE, Döring A, Persson S (2014). The cell biology of cellulose synthesis. Annu Rev Plant Biol.

[29] Kumar M, Turner S (2015). Plant cellulose synthesis: CESA proteins crossing kingdoms. Phytochemistry.

[30] Guerriero G, Fugelstad J, Bulone V (2010). What do we really know about cellulose biosynthesis in higher plants?. J Integr Plant Biol.

[31] Betancur L, Singh B, Rapp RA, Wendel JF, Marks MD, Roberts AW, et al. Phylogenetically distinct cellulose synthase genes support secondary wall thickening in Arabidopsis shoot trichomes and cotton fiber. J Integr Plant Biol. 2010;52:205–20. 10.1111/j.1744-7909.2010.00934.x 20377682

[32] Li A, Xia T, Xu W, Chen T, Li X, Fan J, et al. An integrative analysis of four CESA isoforms specific for fiber cellulose production between *Gossypium hirsutum* and *Gossypium barbadense*. Planta. 2013;237:1585–97. 10.1007/s00425-013-1868-2 23508664

[33] Tanaka K, Murata K, Yamazaki M, Onosato K, Miyao A, Hirochika H (2003). Three distinct rice cellulose synthase catalytic subunit genes required for cellulose synthesis in the secondary wall. Plant Physiol.

[34] Kumar M, Thammannagowda S, Bulone V, Chiang V, Han KH, Joshi CP, et al. An update on the nomenclature for the cellulose synthase genes in Populus. Trends Plant Sci. 2009;14:248–54. 10.1016/j.tplants.2009.02.004 19375973

[35] Handakumbura PP, Matos DA, Osmon KS, Harrington MJ, Heo K, Kafle K, et al. Perturbation of *Brachypodium distachyon* CELLULOSE SYNTHASE A4 or 7 results in abnormal cell walls. BMC Plant Biol. 2013;13:131. 10.1186/1471-2229-13-131 PMC3847494 24024469

[36] Pinard D, Mizrachi E, Hefer CA, Kersting AR, Joubert F, Douglas CJ, et al. Comparative analysis of plant carbohydrate active enzymes and their role in xylogenesis. BMC Genomics. 2015;16:402. 10.1186/s12864-015-1571-8 PMC4440533 25994181

[37] MacMillan CP, Mansfield SD, Stachurski ZH, Evans R, Southerton SG (2010). Fasciclin-like arabinogalactan proteins: specialization for stem biomechanics and cell wall architecture in Arabidopsis and Eucalyptus. Plant J.

[38] Zhong R, Burk DH, Morrison WH, Ye Z-H (2002). A kinesin-like protein is essential for oriented deposition of cellulose microfibrils and cell wall strength. Plant Cell.

[39] Brill E, van Thournout M, White RG, Llewellyn D, Campbell PM, Engelen S, et al. A novel isoform of sucrose synthase is targeted to the cell wall during secondary cell wall synthesis in cotton fiber. Plant Physiol. 2011;157:40–54. 10.1104/pp.111.178574 PMC3165887 21757635

[40] Boerjan W, Ralph J, Baucher M (2003). Lignin biosynthesis. Annu Rev Plant Biol.

[41] Voxeur A, Wang Y, Sibout R (2015). Lignification: different mechanisms for a versatile polymer. Curr Opin Plant Biol.

[42] Vanholme R, Morreel K, Ralph J, Boerjan W (2008). Lignin engineering. Curr Opin Plant Biol.

[43] Bonawitz ND, Chapple C (2010). The fenetics of lignin biosynthesis: connecting genotype to phenotype. Annu Rev Genet.

[44] Ralph J, Lundquist K, Brunow G, Lu F, Kim H, Schatz PF, et al. Lignins: natural polymers from oxidative coupling of 4-hydroxyphenyl- propanoids. Phytochem Rev. 2004;3:29–60.

[45] Zhong R, Ye ZH (2009). Transcriptional regulation of lignin biosynthesis. Plant Signal Behav.

[46] Sonbol F-M, Fornalé S, Capellades M, Encina A, Touriño S, Torres J-L, et al. The maize ZmMYB42 represses the phenylpropanoid pathway and affects the cell wall structure, composition and degradability in *Arabidopsis thaliana*. Plant Mol Biol. 2009;70:283–96. 10.1007/s11103-009-9473-2 19238561

[47] Legay S, Lacombe E, Goicoechea M, Brière C, Séguin A, Mackay J, et al. Molecular characterization of EgMYB1, a putative transcriptional repressor of the lignin biosynthetic pathway. Plant Sci. 2007;173:542–9.

[48] Shen H, He X, Poovaiah CR, Wuddineh WA, Ma J, Mann DGJ, et al. Functional characterization of the switchgrass (*Panicum virgatum*) R2R3-MYB transcription factor PvMYB4 for improvement of lignocellulosic feedstocks. New Phytol. 2012;193:121–36. 10.1111/j.1469-8137.2011.03922.x 21988539

[49] Scheller HV, Ulvskov P (2010). Hemicelluloses. Annu Rev Plant Biol.

[50] Meinert MC, Delmer DP (1977). Changes in biochemical composition of the cell wall of the cotton fiber during development. Plant Physiol.

[51] Singh B, Avci U, Eichler Inwood SE, Grimson MJ, Landgraf J, Mohnen D, et al. A specialized outer layer of the primary cell wall joins elongating cotton fibers into tissue-like bundles. Plant Physiol. 2009;150:684–99. 10.1104/pp.109.135459 PMC2689960 19369592

[52] Hernandez-Gomez MC, Runavot JL, Guo X, Bourot S, Benians TA, Willats WG, et al. Heteromannan and heteroxylan cell wall polysaccharides display different dynamics during the elongation and secondary cell wall deposition phases of cotton fiber cell development. Plant Cell Physiol. 2015;56:1786–97. 10.1093/pcp/pcv101 PMC4562070 26187898

[53] Macmillan CP, Birke H, Bedon F, Pettolino FA. Lignin deposition in cotton cells – where is the lignin? J Plant Biochem Physiol 1: e106.

[54] Fan L, Shi W-J, Hu W-R, Hao X-Y, Wang D-M, Yuan H, et al. Molecular and biochemical evidence for phenylpropanoid synthesis and presence of wall-linked phenolics in cotton fibers. J Integr Plant Biol. 2009;51:626–37. 10.1111/j.1744-7909.2009.00840.x 19566641

[55] Tuttle JR, Nah G, Duke MV, Alexander DC, Guan X, Song Q, et al. Metabolomic and transcriptomic insights into how cotton fiber transitions to secondary wall synthesis, represses lignification, and prolongs elongation. BMC Genomics. 2015;16:1–28. 10.1186/s12864-015-1708-9 PMC4482290 26116072

[56] Yoo M-J, Wendel JF (2014). Comparative evolutionary and developmental dynamics of the cotton *Gossypium hirsutum* Fiber transcriptome. PLoS Genet.

[57] Pettolino FA, Walsh C, Fincher GB, Bacic A (2012). Determining the polysaccharide composition of plant cell walls. Nat Protoc.

[58] Fukushima RS, Hatfield RD (2004). Comparison of the acetyl bromide spectrophotometric method with other analytical lignin methods for determining lignin concentration in forage samples. J Agric Food Chem.

[59] Moreira-Vilar FC, RdC S-S, Finger-Teixeira A, DMd O, Ferro AP, da Rocha GJ, et al. The acetyl bromide method is faster, simpler and presents best recovery of lignin in different herbaceous tissues than Klason and thioglycolic acid methods. PLoS One. 2014;9:e110000. 10.1371/journal.pone.0110000 PMC4212577 25330077

[60] Kim H, Ralph J. Solution-state 2D NMR of ball-milled plant cell wall gels in DMSO-d_6_/pyridine-d_5_. Org Biomol Chem. 2010;8:576–91. 10.1039/b916070a PMC4070321 20090974

[61] Paterson AH, Wendel JF, Gundlach H, Guo H, Jenkins J, Jin D, et al. Repeated polyploidization of Gossypium genomes and the evolution of spinnable cotton fibres. Nature. 2012;492:423–7. 10.1038/nature11798 23257886

[62] Goodstein DM, Shu S, Howson R, Neupane R, Hayes RD, Fazo J, et al. Phytozome: a comparative platform for green plant genomics. Nucleic Acids Res. 2012;40:D1178–D86. https://phytozome.jgi.doe.gov/pz/portal.html 10.1093/nar/gkr944 PMC3245001 22110026

[63] Kim D, Pertea G, Trapnell C, Pimentel H, Kelley R, Salzberg SL (2013). TopHat2: accurate alignment of transcriptomes in the presence of insertions, deletions and gene fusions. Genome Biol.

[64] Trapnell C, Hendrickson DG, Sauvageau M, Goff L, Rinn JL, Pachter L (2013). Differential analysis of gene regulation at transcript resolution with RNA-seq. Nat Biotechnol.

[65] Quinlan AR, Hall IM (2010). BEDTools: a flexible suite of utilities for comparing genomic features. Bioinformatics.

[66] Robinson MD, Oshlack A (2010). A scaling normalization method for differential expression analysis of RNA-seq data. Genome Biol.

[67] Nookaew I, Papini M, Pornputtapong N, Scalcinati G, Fagerberg L, Uhlén M, et al. A comprehensive comparison of RNA-Seq-based transcriptome analysis from reads to differential gene expression and cross-comparison with microarrays: a case study in *Saccharomyces cerevisiae*. Nucleic Acids Res. 2012;40:10084–97. 10.1093/nar/gks804 PMC3488244 22965124

[68] Reimand J, Arak T, Vilo J (2011). G:Profiler—a web server for functional interpretation of gene lists (2011 update). Nucleic Acids Res.

[69] Reimand J, Kull M, Peterson H, Hansen J, Vilo J (2007). G:Profiler—a web-based toolset for functional profiling of gene lists from large-scale experiments. Nucleic Acids Res.

[70] Benjamini Y, Hochberg Y (1995). Controlling the false discovery rate - a practical and powerful approach to multiple testing. J Roy Stat Soc B Met.

[71] Maltby D, Carpita NC, Montezinos D, Carl K, Delmer DP (1979). B-1,3-Glucan in developing cotton fibers. Structure, localization, and relationship of synthesis to that of secondary wall cellulose. Plant Physiol.

[72] Musel G, Schindler T, Bergfeld R, Ruel K, Jacquet G, Lapierre C, et al. Structure and distribution of lignin in primary and secondary cell walls of maize coleoptiles analyzed by chemical and immunological probes. Planta. 1997;201:146–59.

[73] Christiernin M, Ohlsson AB, Berglund T, Henriksson G (2005). Lignin isolated from primary walls of hybrid aspen cell cultures indicates significant differences in lignin structure between primary and secondary cell wall. Plant Physiol Biochem.

[74] Lamesch P, Berardini TZ, Li D, Swarbreck D, Wilks C, Sasidharan R, et al. The Arabidopsis information resource (TAIR): improved gene annotation and new tools. Nucleic Acids Res. 2012;40:D1202–D10. www.arabidopsis.org 10.1093/nar/gkr1090 PMC3245047 22140109

[75] Hussey S, Mizrachi E, Spokevicius A, Bossinger G, Berger D, Myburg A (2011). SND2, a NAC transcription factor gene, regulates genes involved in secondary cell wall development in Arabidopsis fibres and increases fibre cell area in Eucalyptus. BMC Plant Biol.

[76] Zhao CS, Avci U, Grant EH, Haigler CH, Beers EP (2008). XND1, a member of the NAC domain family in *Arabidopsis thaliana*, negatively regulates lignocellulose synthesis and programmed cell death in xylem. Plant J.

[77] Öhman D, Demedts B, Kumar M, Gerber L, Gorzsás A, Goeminne G, et al. MYB103 is required for FERULATE-5-HYDROXYLASE expression and syringyl lignin biosynthesis in Arabidopsis stems. Plant J. 2013;73:63–76. 10.1111/tpj.12018 22967312

[78] Zhou J, Lee C, Zhong R, Ye Z-H (2009). MYB58 and MYB63 are transcriptional activators of the lignin biosynthetic pathway during secondary cell wall formation in Arabidopsis. Plant Cell.

[79] Ko J-H, Jeon H-W, Kim W-C, Kim J-Y, Han K-H (2014). The MYB46/MYB83-mediated transcriptional regulatory programme is a gatekeeper of secondary wall biosynthesis. Ann Bot.

[80] Bhargava A, Ahad A, Wang S, Mansfield S, Haughn G, Douglas C, et al. The interacting MYB75 and KNAT7 transcription factors modulate secondary cell wall deposition both in stems and seed coat in Arabidopsis. Planta. 2013;237:1199–211. 10.1007/s00425-012-1821-9 23328896

[81] Wang H, Avci U, Nakashima J, Hahn MG, Chen F, Dixon RA (2010). Mutation of WRKY transcription factors initiates pith secondary wall formation and increases stem biomass in dicotyledonous plants. Proc Natl Acad Sci U S A.

[82] Ding M, Chen J, Jiang Y, Lin L, Cao Y, Wang M, et al. Genome-wide investigation and transcriptome analysis of the WRKY gene family in Gossypium. Mol Gen Genomics. 2015;290:151–71. 10.1007/s00438-014-0904-7 25190108

[83] Nawy T, Lee J-Y, Colinas J, Wang JY, Thongrod SC, Malamy JE, et al. Transcriptional profile of the Arabidopsis root quiescent center. Plant Cell. 2005;17:1908–25. 10.1105/tpc.105.031724 PMC1167541 15937229

[84] Goubet F, Barton CJ, Mortimer JC, Yu X, Zhang Z, Miles GP, et al. Cell wall glucomannan in Arabidopsis is synthesised by CSLA glycosyltransferases, and influences the progression of embryogenesis. Plant J. 2009;60:527–38. 10.1111/j.1365-313X.2009.03977.x 19619156

[85] Zabotina OA (2012). Xyloglucan and its biosynthesis. Front Plant Sci.

[86] Verhertbruggen Y, Yin L, Oikawa A, Scheller HV (2011). Mannan synthase activity in the CSLD family. Plant Signal Behav.

[87] Kumar S, Kumar K, Pandey P, Rajamani V, Padmalatha KV, Dhandapani G, et al. Glycoproteome of elongating cotton fiber cells. Mol Cell Proteomics. 2013;12:3677–89. 10.1074/mcp.M113.030726 PMC3861716 24019148

[88] Hu G, Koh J, Yoo M-J, Grupp K, Chen S, Wendel JF (2013). Proteomic profiling of developing cotton fibers from wild and domesticated *Gossypium barbadense*. New Phytol.

[89] Tan J, Tu L, Deng F, Hu H, Nie Y, Zhang X. A genetic and metabolic analysis revealed that cotton fiber cell development was retarded by flavonoid naringenin. Plant Physiol. 2013;162:86–95. 10.1104/pp.112.212142 PMC3641232 23535943

