# Correction to: Tissue and cell-specific transcriptomes in cotton reveal the subtleties of gene regulation underlying the diversity of plant secondary cell walls

**DOI:** 10.1186/s12864-018-4634-9

**Published:** 2018-04-17

**Authors:** Colleen P. MacMillan, Hannah Birke, Aaron Chuah, Elizabeth Brill, Yukiko Tsuji, John Ralph, Elizabeth S. Dennis, Danny Llewellyn, Filomena A. Pettolino

**Affiliations:** 1 CSIRO Agriculture and Food, PO Box 1700, Canberra, ACT 2601 Australia; 2 Present address: Research School of Biology, The Australian National University, Canberra, ACT 2601 Australia; 3 John Curtin School of Medical Research, The Australian National University, ACT, Canberra, 2601 Australia; 4 Department of Biochemistry and the Department of Energy’s Great Lakes BioEnergy Research Center, The Wisconsin Energy Institute, 1552 University Avenue, Madison, WI 53726-4084 USA

## Correction

Upon publication of the original article [1], the authors had flagged that Fig. 1 had been published twice, as both Fig. 1 and Additional file 3.

The updated Additional file 3 (Corrected Additional file 3, is included with this Correction).

## Additional file

Additional file 3: Partial HSQC spectra of cotton seed fibres, along with those from a synthetic lignin and Arabidopsis thaliana. (PDF 8149 kb)
