# Supplementary material for: Tissue and cell-specific transcriptomes in cotton reveal the subtleties of gene regulation underlying the diversity of plant secondary cell walls
Source: BMC Genomics. 2017 Jul 18;18:539. doi: 10.1186/s12864-017-3902-4 (PMC5516393; doi:10.1186/s12864-017-3902-4)
Supplement: Supplementary file 1 — Cotton stem pith and xylem used in RNA extraction for deep-sequencing. (PDF 193 kb) [file 12864_2017_3902_MOESM1_ESM.pdf]

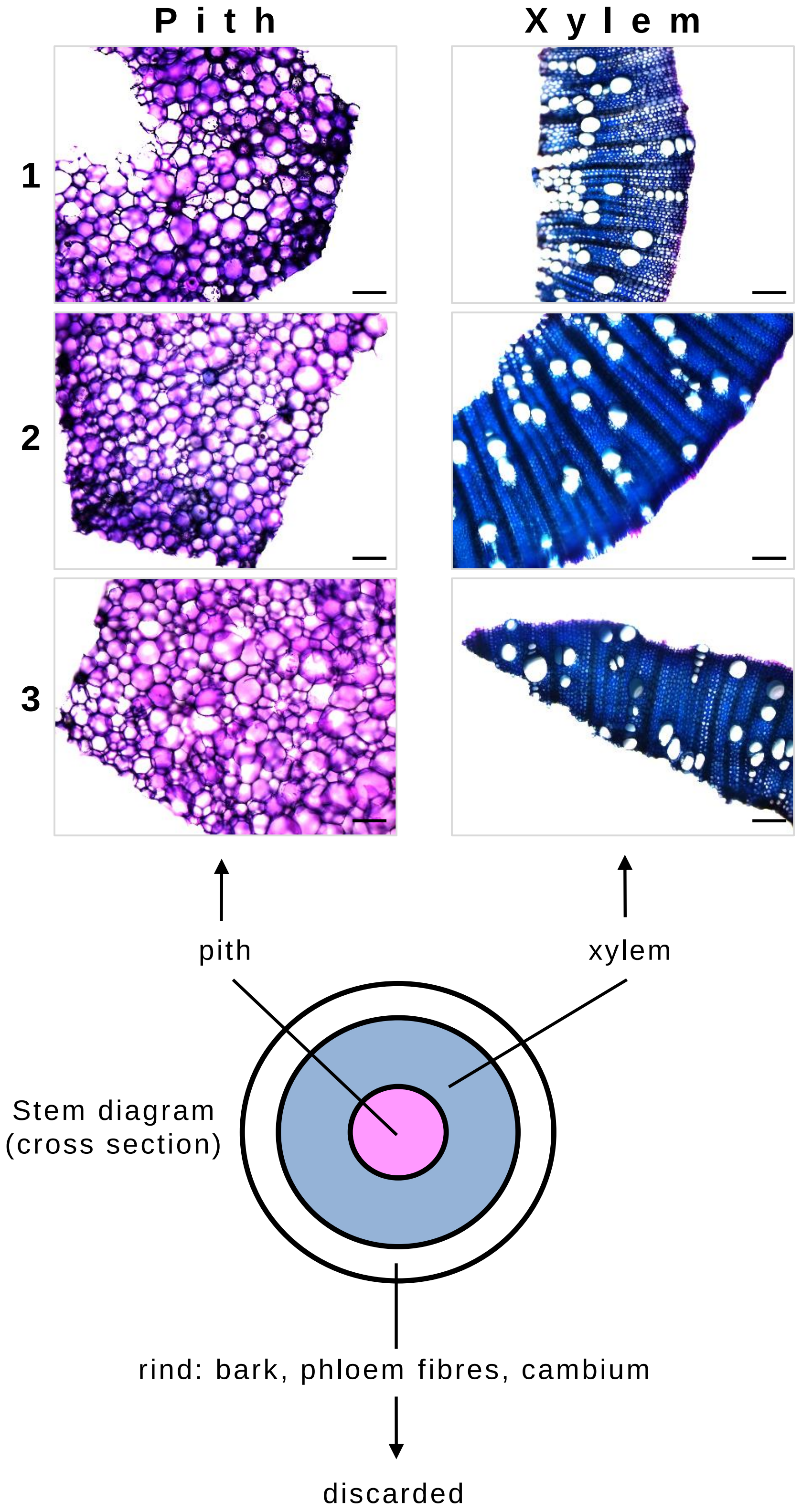

Additional file 1 Cotton stem pith and xylem used in RNA extraction for deep-sequencing. Following dissection of stem-specific tissues (pith, xylem) for RNA extraction, cross-sections were made and fixed in 70 % ethanol (v/v), then stained with toluidine. Number refers to biological replicate. Bar = 50 micrometers.
