# Supplementary material for: Tissue and cell-specific transcriptomes in cotton reveal the subtleties of gene regulation underlying the diversity of plant secondary cell walls
Source: BMC Genomics. 2017 Jul 18;18:539. doi: 10.1186/s12864-017-3902-4 (PMC5516393; doi:10.1186/s12864-017-3902-4)
Supplement: Supplementary file 2 — Primer sequences for qPCR. (PDF 80 kb) [file 12864_2017_3902_MOESM2_ESM.pdf]

Gene	Primer	Sequence (5’ → 3”)
Ubiquitin	UBQ_for	TTTGCACCTGGTCCTTCG
	UBQ_rev	GCTTCCCCTGCCTAGTGG
Gorai.007G112500	NST1a_for	ATCTCCATGGGCTACAGCAA
	NST1a_rev	CGATGAGGATGATCTGGTGA
Gorai.008G130300	NST1b_for	CCCTATTAGCACATCCTTGTCTG
	NST1b_rev	GACATGACAAATCTCGTGTTGTT
Gorai.003G077700	SND1a_for	GAAGACGGTGAAGACGATGACT
	SND1a_rev	TGACCCTAATGCGGTTTTTAGT
Gorai.008G259700	SND1b_for	GAGAATGGTTCATTGAGCAACA
	SND1b_rev	TCGATGGAGTGGTAACAACAAG

Additional file 2 Primer sequences for qPCR. Nucleotide sequences of primers used for qPCR are shown. Annealing temperature of all primers was 60 °C. for, forward; rev, reverse.
