# Supplementary material for: Tissue and cell-specific transcriptomes in cotton reveal the subtleties of gene regulation underlying the diversity of plant secondary cell walls
Source: BMC Genomics. 2017 Jul 18;18:539. doi: 10.1186/s12864-017-3902-4 (PMC5516393; doi:10.1186/s12864-017-3902-4)
Supplement: Supplementary file 3 — Partial HSQC spectra of cotton seed fibres, along with those from a synthetic lignin and Arabidopsis thaliana. (PDF 134 kb) [file 12864_2017_3902_MOESM3_ESM.pdf]

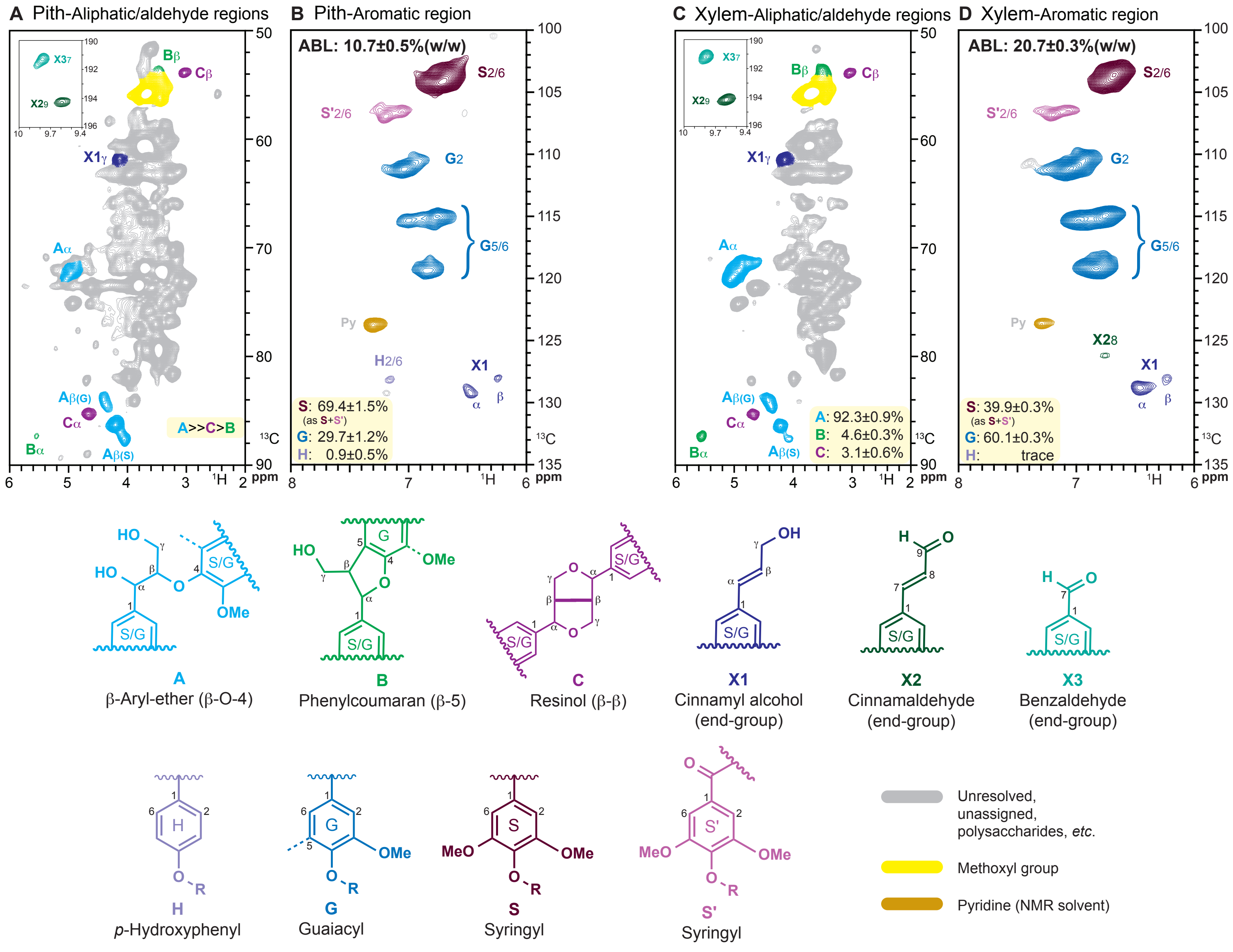

A Pith-Aliphatic/aldehyde regions
B Pith-Aromatic region
C Xylem-Aliphatic/aldehyde regions
D Xylem-Aromatic region
ABL: 10.7±0.5%(w/w)
ABL: 20.7±0.3%(w/w)
A>>C>B
S: 69.4±1.5% (as S+S')
G: 29.7±1.2%
H: 0.9±0.5%
A: 92.3±0.9%
B: 4.6±0.3%
C: 3.1±0.6%
S: 39.9±0.3% (as S+S')
G: 60.1±0.3%
H: trace
Aα Aβ(G) Aβ(S) Bα Bβ Cα Cβ X1γ X37 X29 X28 X1 α β S2/6 S'2/6 G2 G5/6 Py H2/6
^{13}C ^{1}H ppm
A
β-Aryl-ether (β-O-4)
B
Phenylcoumaran (β-5)
C
Resinol (β-β)
X1
Cinnamyl alcohol (end-group)
X2
Cinnamaldehyde (end-group)
X3
Benzaldehyde (end-group)
H
p-Hydroxyphenyl
G
Guaiacyl
S
Syringyl
S'
Syringyl
Unresolved, unassigned, polysaccharides, etc.
Methoxyl group
Pyridine (NMR solvent)
