# Supplementary material for: Tissue and cell-specific transcriptomes in cotton reveal the subtleties of gene regulation underlying the diversity of plant secondary cell walls
Source: BMC Genomics. 2017 Jul 18;18:539. doi: 10.1186/s12864-017-3902-4 (PMC5516393; doi:10.1186/s12864-017-3902-4)
Supplement: Supplementary file 4 — Monosaccharide linkage composition of cell walls. (PDF 153 kb) [file 12864_2017_3902_MOESM4_ESM.pdf]

Monosaccharide	Deduced linkage	Fibre 7	sd	Fibre 15	sd	Fibre 25	sd	Pith	sd	Xylem	sd
Rha(p)	terminal	0.6[b]	0.3	0.5	0.1	tr	0.0	tr	0.0	0.1	0.0
	1,2-	1.4	0.4	1.7	0.2	0.2	0.0	0.2	0.0	0.1	0.1
	1,2,4-	0.7	0.3	0.7	0.1	tr	0.0	0.2	0.0	0.2	0.0
	1,2,3,4,6-	0.1	0.1	0.1	0.1	-	0.0	-	0.0	-	0.0
Fuc(p)	terminal	0.4	0.0	0.4	0.0	tr	0.0	tr	0.0	0.1	0.1
Ara(f)	terminal	6.2	0.3	4.6	0.0	0.3	0.1	0.2	0.1	0.2	0.1
	1,2-	0.4	0.1	0.4	0.0	0.1	0.0	-	0.0	-	0.0
	1,3-	0.1	0.0	0.1	0.0	tr	0.0	-	0.0	-	0.0
	1,5-	13.0	0.4	7.8	0.1	0.2	0.1	0.7	0.1	0.5	0.2
	1,2,5-	1.1	0.7	0.4	0.0	-	0.0	tr	0.0	-	0.0
	1,2,3,5-	0.6	0.6	0.1	0.2	-	0.0	-	0.0	-	0.0
Ara(p)	terminal	0.2	0.0	0.2	0.1	tr	0.0	tr	0.0	tr	0.0
Xyl(p)	terminal	2.5	0.3	2.3	0.2	0.2	0.1	0.5	0.1	0.5	0.1
	1,2-	0.5	0.1	0.6	0.1	0.1	0.0	1.8	0.6	2.2	0.3
	1,4-	2.1	0.6	1.3	0.2	0.5	0.1	16.2	5.8	20.1	2.1
	1,2,4-	0.3	0.2	0.2	0.0	0.1	0.0	1.8	0.7	1.9	0.1
	1,3,4-	-	0.0	-	0.0	-	0.0	-	0.0	0.6	0.2
	1,2,3,4-	0.3	0.2	tr	0.0	-	0.0	tr	0.0	1.6	1.8
Man(p)	1,4-	1.8	0.3	1.6	0.2	0.6	0.0	1.4	0.0	1.7	0.3
	1,4,6-	0.5	0.1	0.6	0.0	tr	0.0	-	0.0	-	0.0
t-Gal(p)	terminal	2.0	0.0	2.2	0.1	0.2	0.0	0.3	0.0	0.2	0.0
	1,2-	0.7	0.3	1.0	0.1	0.1	0.0	0.1	0.0	0.2	0.0
	1,3-	0.6	0.0	0.5	0.0	0.1	0.0	tr	0.0	tr	0.0
	1,4-	1.8	0.4	1.5	0.2	tr	0.0	0.5	0.1	0.3	0.0
	1,6-	1.1	0.3	1.0	0.1	0.1	0.0	0.2	0.0	0.3	0.1
	1,3,4-	0.3	0.0	0.4	0.0	tr	0.0	tr	0.0	-	0.0
	1,3,6-	2.8	0.1	3.1	0.1	0.3	0.0	0.1	0.0	0.1	0.0
	1,4,6-	0.3	0.1	0.3	0.1	-	0.0	-	0.0	-	0.0
	1,3,4,6-	0.1	0.1	tr	0.1	-	0.0	-	0.0	-	0.0
	1,2,3,4,6-	1.2	1.8	0.2	0.3	-	0.0	-	0.0	-	0.0
Glc(p)	terminal	0.5	0.0	1.6	0.2	2.2	0.3	2.4	0.4	0.7	0.2
	1,3-	2.0	0.5	11.4	0.9	8.3	2.4	0.1	0.0	0.1	0.0
	1,4-	25.8	0.6	30.9	0.7	82.0	2.8	65.0	6.8	60.7	5.9
	1,2,3-	-	0.0	0.1	0.0	0.1	0.0	0.1	0.1	-	0.0
	1,2,4-	0.3	0.1	0.4	0.1	0.5	0.1	0.3	0.2	0.6	0.2
	1,3,4-	0.6	0.1	0.5	0.0	0.9	0.1	0.6	0.0	1.3	0.1
	1,3,6-	0.1	0.0	0.8	0.0	0.4	0.1	-	0.0	-	0.0
	1,4,6-	3.2	0.4	3.6	0.3	1.2	0.3	0.9	0.3	0.8	0.1
	1,2,3,4-	-	0.0	-	0.0	0.2	0.0	0.2	0.0	0.2	0.0
	1,2,4,6-	-	0.0	-	0.0	tr	0.0	-	0.0	-	0.0
	1,3,4,6-	0.2	0.1	0.2	0.2	tr	0.0	-	0.0	0.2	0.2
	1,2,3,4,6-	0.5	0.7	0.1	0.1	tr	0.0	-	0.0	0.3	0.5
Glc A(p)	terminal	1.4	0.3	0.7	0.1	0.1	0.1	1.6	0.2	2.5	0.2
GalA(p)	terminal	0.6	0.1	0.5	0.1	0.1	0.0	0.1	0.0	0.1	0.1
	1,4-	21.2 (49.0)[c]	4.5 (5.7)	15.6 (42.5)	0.5 (3.5)	0.8 (20.0)	0.4 (0)	4.3 (51.0)	0.6 (5.7)	1.2 (42.5)	0.1 (3.5)

[a]Determined from analysis of duplicate samples; [b]Deduced from 1,5-di-O-acetyl-6-deoxy-2,3,4-tri-O-methyl hexitol; [c]values in parentheses represent degree of esterification; -, not detected; tr, <0.05 mol%

Additional file 4 Monosaccharide linkage composition of cell walls (mol%)[a] used to estimate relative polysaccharide composition of fibre and stem cell walls as presented in Table 1.
