# Supplementary material for: Tissue and cell-specific transcriptomes in cotton reveal the subtleties of gene regulation underlying the diversity of plant secondary cell walls
Source: BMC Genomics. 2017 Jul 18;18:539. doi: 10.1186/s12864-017-3902-4 (PMC5516393; doi:10.1186/s12864-017-3902-4)
Supplement: Supplementary file 5 — Cotton stem cell cross-sections stained with toluidine blue and Maule reaction. (PDF 361 kb) [file 12864_2017_3902_MOESM5_ESM.pdf]

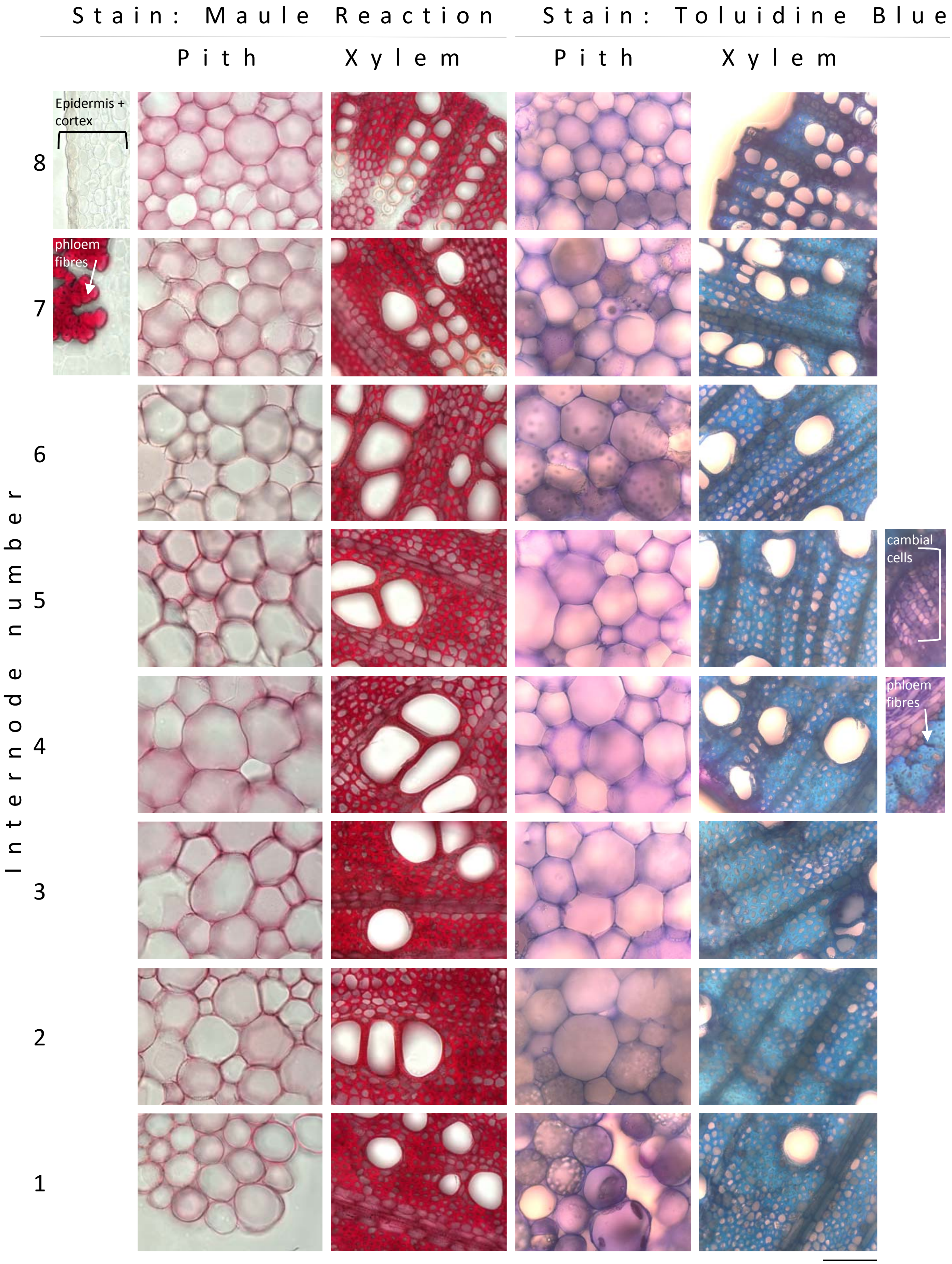

Additional file 5. Cotton stem cell cross-sections. Individual internodes were fixed in 70% ethanol (v/v) before sectioning to ~ 100 micron thickness. Cross sections were stained via Maule reaction for lignin - indicated by red / brown, or toluidine blue for cell walls where generally primary cell walls stain pink/purple and secondary walls with cellulose and polyphenolics stain blue/green. Internode number starts from stem-base of a ~ 16-week old Coker315-11 cotton plant. Bar = 50 microns.
