# Supplementary material for: Tissue and cell-specific transcriptomes in cotton reveal the subtleties of gene regulation underlying the diversity of plant secondary cell walls
Source: BMC Genomics. 2017 Jul 18;18:539. doi: 10.1186/s12864-017-3902-4 (PMC5516393; doi:10.1186/s12864-017-3902-4)
Supplement: Supplementary file 7 — Phylogenetic tree of NAC TFs differentially expressed during cotton SCW development. (PDF 1214 kb) [file 12864_2017_3902_MOESM7_ESM.pdf]

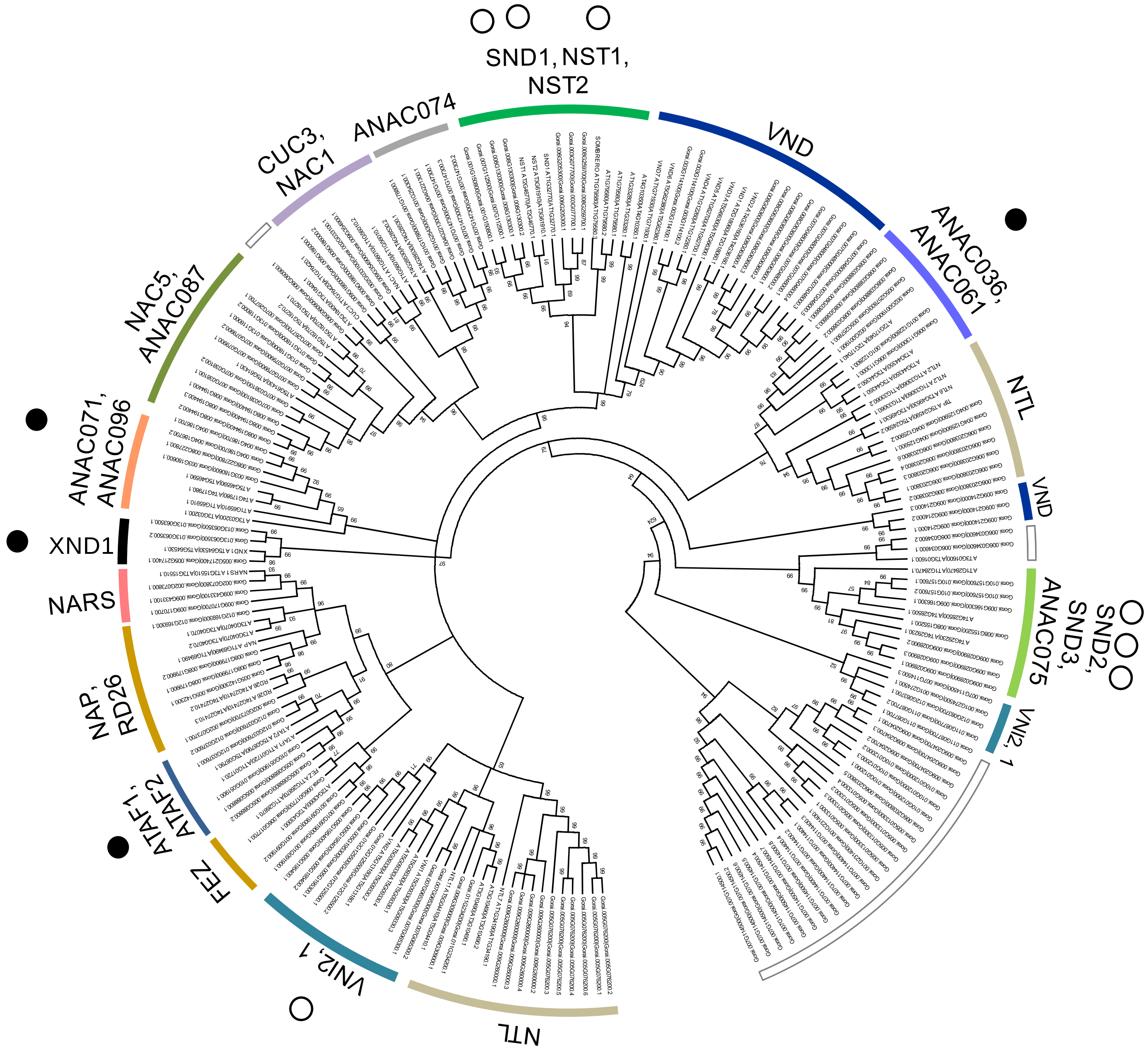

Additional file 7 Phylogenetic tree of NAC TFs differentially expressed during cotton SCW development. Phylogenetic comparison of all the differentially expressed cotton NACs with their closest Arabidopsis counterparts. The consensus neighbour-joining tree was generated in MEGA 6 using a MUSCLE alignment of all the D-genome (*G. raimondii)* protein sequences of NACs differentially expressed in cotton PCW and SCW tissues along with known Arabidopsis NACs; all known splice variants for each gene were included. Refer to Fig. 3 for details of the differentially expressed genes. Coloured arcs indicate different NAC groups that are labelled with Arabidopsis NAC-gene identifiers. Uncoloured arcs indicate 'other NACs'. The white dots highlight NACs that are particularly highly upregulated in highly cellulosic SCWs (seed fibres) compared to xylem, and the black dots indicate NACs particularly high upregulated in lignocellulosic SCWs (xylem) compared to that in seed fibres.
