# Supplementary material for: Tissue and cell-specific transcriptomes in cotton reveal the subtleties of gene regulation underlying the diversity of plant secondary cell walls
Source: BMC Genomics. 2017 Jul 18;18:539. doi: 10.1186/s12864-017-3902-4 (PMC5516393; doi:10.1186/s12864-017-3902-4)
Supplement: Supplementary file 8 — Other TFs expressed during Gossypium hirsutum SCW and PCW formation. (PDF 30 kb) [file 12864_2017_3902_MOESM8_ESM.pdf]

A

Auxin-rel TF group	Gorai Gene ID	XYLM	PITH	SF07	SF15	SF25
AUX2-11, IAA4	013G200800	219	258	24	54	18
	004G075000	196	90	14	15	1
	001G242800	115	29	42	8	0
	005G046400	23	41	4	2	1
	006G245900	7	1	21	14	0
	011G170700	5	2	0	0	0
	007G277000	2	0	24	11	2
AXR2, IAA7	001G242900	120	38	137	25	16
IAA11	001G181800	30	24	6	5	4
	004G222500	29	67	14	16	19
	007G150000	67	56	18	12	17
IAA13	006G250900	30	5	2	1	1
	011G180100	316	45	7	11	18
IAA14, SLR	005G046500	8	75	2	2	1
	006G246000	0	0	33	226	15
IAA16	007G276900	70	43	4	4	2
	013G200700	186	149	8	8	7
IAA18	009G048400	36	4	1	1	1
IAA19, MSG2	010G227800	11	2	2	1	0
IAA29	009G016200	8	2	18	23	5
	009G058600	9	49	3	3	4
IAA30	007G117700	0	0	1	124	5
IAA9	010G031600	1387	1280	470	414	225
	009G226000	629	577	274	285	247
	003G035700	97	93	52	37	39
	011G109000	37	61	102	87	37
ARF7, TIR5*	009G037100	154	66	39	24	47
ARF8	001G204500	48	78	26	22	19
	006G008700	73	64	18	19	26
	008G054600	41	39	29	21	14
	008G097200	82	81	21	22	28
	009G152700	51	9	27	15	14
	011G048200	63	35	37	30	17
ARF11	001G153900	177	180	70	44	40
ARF18	007G109500	63	112	75	41	15
	008G126200	26	60	190	129	21
ARF1-BP, ARF2	001G054600	301	254	81	73	89
ARF4	006G120400	75	31	27	16	13
	009G078800	31	4	4	0	2
ARF9	003G078000	254	249	21	13	24
	007G026900	199	99	75	39	25
ARF3, ETT	009G166100	106	120	68	95	35
	010G157400	21	41	10	7	6
	011G030900	124	120	26	31	28
ARF10	003G142500	58	96	26	57	52
ARF16	011G238900	24	9	91	72	51
ARF11/19, IAA22	001G017000	243	184	56	65	62
	heat-map colour-key	*0*	*25*	*50*	*75*	*100+*

B

BEL1-like group	Gorai Gene ID	XYLM	PITH	SF07	SF15	SF25
BLH1, EDA29	009G396900	205	272	25	86	147
	002G234900	297	412	11	34	66
	006G149100	154	225	9	31	59
	006G115600	91	427	3	11	21
	009G082700	52	155	1	5	15
	001G125400	80	83	3	29	15
BLH2, SAW1	007G207200	113	61	1	3	90
	008G070800	29	28	0	0	47
	003G018100	50	13	1	1	29
BLH6	013G034600	48	76	11	38	122
	008G031100	49	42	10	9	26
	005G246400	60	67	13	4	10
BLH7	010G007800	30	50	2	4	22
BLH8, PNF	009G109300	23	26	0	0	8
	heat-map colour-key	*0*	*25*	*50*	*75*	*100+*

C

KNAT group	Gorai Gene ID	XYLM	PITH	SF07	SF15	SF25
KNAT1	005G098100	6	11	0	0	0
	009G223200	197	276	0	1	2
	010G029000	377	184	0	0	0
KNAT2	007G306500	5	39	0	0	0
KNAT3	001G035700	45	79	16	10	9
	010G117100	69	63	4	4	3
KNAT6	004G236400	5	24	2	3	0
	005G180500	19	27	2	1	0
	008G296800	10	10	0	0	0
	009G336900	12	28	0	0	0
	013G129400	49	43	1	1	1
KNAT7	003G163800	46	12	18	44	96
	004G206600	80	52	51	127	294
	008G242800	48	13	16	14	40
	heat-map colour-key	*0*	*75*	*150*	*225*	*300+*

D

bHLH Gorai Gene ID	XYLM	PITH	SF07	SF15	SF25
001G242500	17	1	2	1	0
002G016500	14	38	39	92	101
002G228900	8	9	2	1	1
002G229100	4	3	12	24	13
003G009400	90	51	6	7	8
003G060900	0	0	1	11	1
003G182100	23	49	4	8	13
004G075200	62	78	17	17	17
004G184800	913	420	35	24	19
004G215900	11	14	64	74	64
004G218900	52	30	7	7	8
004G243100	35	22	1	1	1
005G022400	18	3	91	103	14
005G136000	23	5	0	0	0
005G148300	34	15	18	10	9
006G021800	0	0	24	11	1
006G140200	6	7	1	1	1
006G216700	60	32	8	7	7
007G023500	24	15	2	0	1
007G040600	96	127	14	16	15
007G088300	18	16	0	0	0
007G109300	107	141	22	19	18
007G111800	1	12	9	20	7
007G113200	0	0	57	32	24
007G113300	0	0	54	32	26
007G136400	12	14	43	59	40
007G157700	13	2	8	15	11
007G194400	0	0	9	4	1
007G277200	222	129	3	11	9
007G352300	18	4	3	2	1
007G361700	1	0	56	43	5
008G067500	10	8	3	2	20
008G106900	25	6	0	0	0
008G128800	3	18	0	0	0
008G154300	59	7	0	0	0
008G159000	0	1	12	15	11
008G186700	69	4	12	10	5
008G226300	76	51	11	12	19
009G002200	6	6	0	0	0
009G071900	54	52	1	0	1
009G176000	50	32	27	35	129
009G213800	1	4	9	12	4
010G028200	55	56	45	43	18
011G034000	144	23	2	3	83
011G144600	59	28	5	4	8
011G150200	17	5	25	28	13
011G172500	13	14	3	3	6
011G292100	107	36	4	3	2
012G055200	0	0	8	0	0
012G127900	54	9	0	0	0
013G079600	59	1	3	4	6
013G098600	20	18	20	10	4
013G138300	14	14	0	1	1
013G242800	81	23	1	1	1
heat-map colour-key	*0*	*50*	*100*	*150*	*200+*

Additional file 8 Other TFs expressed during *Gossypium hirsutum* SCW and PCW formation. (A) Auxin-related, (B) BEL1-like-related, (C) KNAT-related, (D) bHLH-related.
