# Supplementary material for: Tissue and cell-specific transcriptomes in cotton reveal the subtleties of gene regulation underlying the diversity of plant secondary cell walls
Source: BMC Genomics. 2017 Jul 18;18:539. doi: 10.1186/s12864-017-3902-4 (PMC5516393; doi:10.1186/s12864-017-3902-4)
Supplement: Supplementary file 9 — Other cell wall related genes that were differentially expressed across the cotton tissues. (PDF 17 kb) [file 12864_2017_3902_MOESM9_ESM.pdf]

cell wall-related group	Gorai Gene ID	TISSUE XYLM	PITH	SF07	SF15	SF25
ABI8	*013G014900*	42	39	103	144	301
BGLU42	*003G088100*	20	25	72	82	63
BOT1	*006G100400*	25	27	39	76	130
COB	*008G200000*	22	9	195	127	89
COBL1	*001G208300*	54	41	30	45	16
COBL2	*008G033200*	0	0	14	9	0
COBL4	*003G102500*	24	0	0	0	1
	004G063600	23	3	1	8	1661
	007G176400	45	3	2	12	1110
	008G200100	3	0	1	1	43
CSI1	*007G242400*	130	73	308	578	739
CTL1	*011G005100*	113	98	580	1035	1230
CTL2	*006G078900*	289	16	6	11	8034
	011G198500	483	25	5	7	11349
FRA1	*002G134500*	43	46	57	124	1127
	003G026200	128	23	522	437	21
	009G214600	116	28	112	75	58
HCHIB	*010G058900*	0	0	0	5	47
	005G257900	11	4	28	10	4
HERK1	*001G107000*	11	16	88	56	196
	003G137700	6	3	99	80	20
HERK2	*009G115900*	7	3	48	41	33
IRX9	*006G168500*	30	3	22	198	875
IRX9-L	*005G097300*	14	15	24	21	46
	010G031000	3	2	37	50	112
IRX10	*005G197500*	79	4	24	120	2043
	008G156000	15	1	1	3	7
IRX14-L	*003G026800*	45	14	7	10	18
	007G216800	62	29	16	10	20
KINESIN-13A	*006G262200*	35	32	86	82	128
KOR1	*010G143300*	200	35	285	589	8069
KOR2	*011G089500*	53	19	74	144	488
PARVUS	*009G205300*	25	7	1	1	7
	009G274300	47	0	0	0	1
RSW3	*007G066900*	52	62	255	192	139
	heat-map colour-key	*0*	*125*	*250*	*375*	*500+*

Additional file 9 Other cell wall related genes that were differentially expressed across the cotton tissues.
