# Supplementary material for: Correction to: Tissue and cell-specific transcriptomes in cotton reveal the subtleties of gene regulation underlying the diversity of plant secondary cell walls
Source: BMC Genomics. 2018 Apr 17;19:261. doi: 10.1186/s12864-018-4634-9 (PMC5902885; doi:10.1186/s12864-018-4634-9)
Supplement: Supplementary file 1 — Partial HSQC spectra of cotton seed fibres, along with those from a synthetic lignin and Arabidopsis thaliana. (PDF 8149 kb) [file 12864_2018_4634_MOESM1_ESM.pdf]

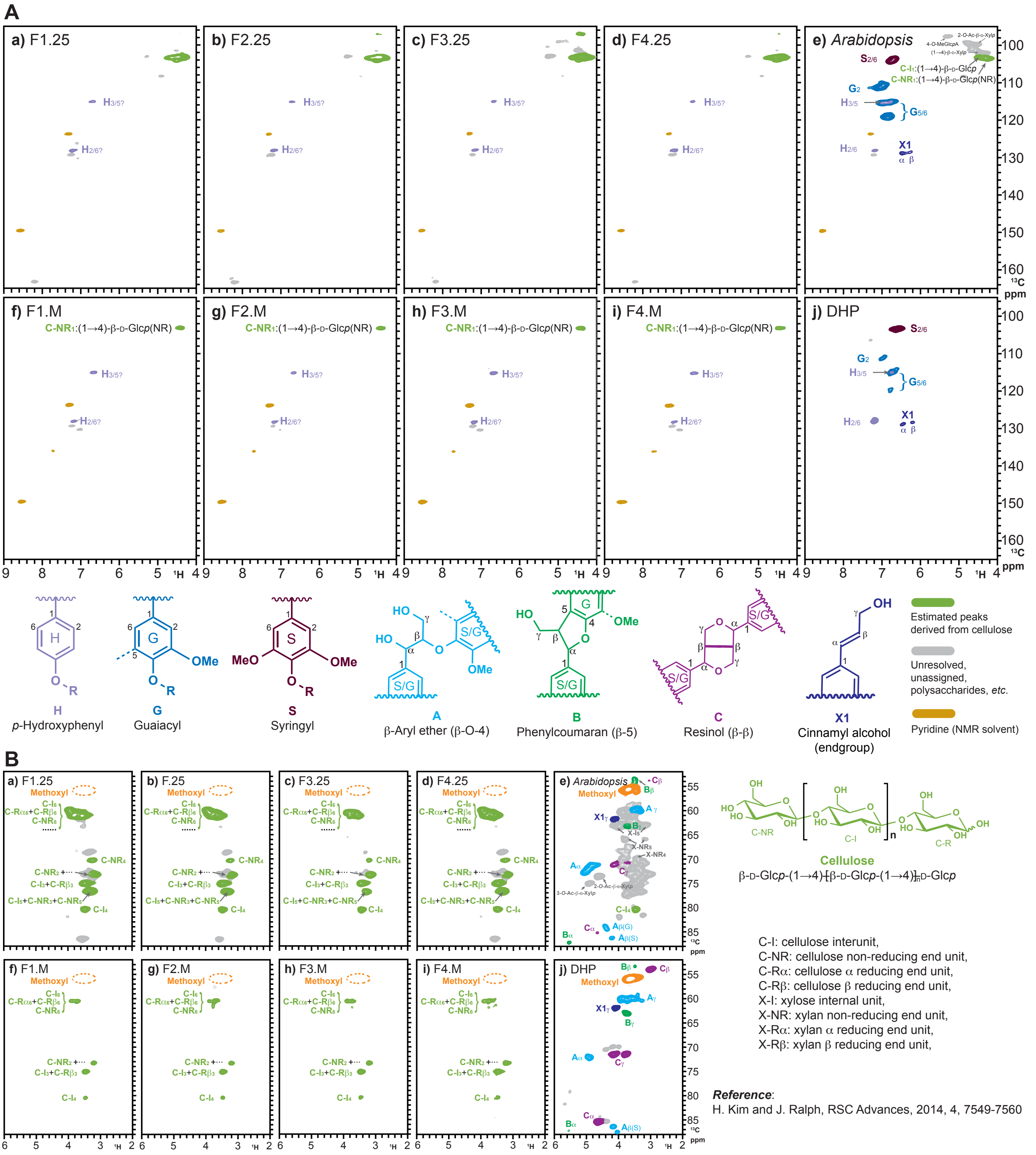

Additional file 3 Partial HSQC spectra of cotton seed fibres, along with those from a synthetic lignin and *Arabidopsis thaliana*. Aromatic/anomeric subregion (A) and aliphatic subregion (B). 25 DPA (a-d) and matured (f-i) cotton seed fibres were compared with Arabidopsis (e) having a typical dicot plant lignin and polysaccharides, and with a DHP (j) prepared from *p*-coumaryl alcohol, coniferyl alcohol and sinapyl alcohol (ratio: 1;4:4). Cotton seed fibres were harvested from four plants (F1-F4) individually. It is clear from the comparison with *Arabidopsis* (e) and DHP (j) that seed fibres lack correlation peaks derived from both guaiacyl and syringyl units (A). Evidence that these typical lignin units are not present is also supported by the absence of methoxyl groups in seed fibres (B). The colored contours in the spectra correspond to the chemical structures shown in the same color. Assignments were via comparison with previous data [90].
